# A combinatorial *cis*-regulatory logic restricts color-sensing Rhodopsins to specific photoreceptor subsets in *Drosophila*

**DOI:** 10.1371/journal.pgen.1009613

**Published:** 2021-06-23

**Authors:** Clara Poupault, Diane Choi, Khanh Lam-Kamath, Deepshe Dewett, Ansa Razzaq, Joseph Bunker, Alexis Perry, Irene Cho, Jens Rister

**Affiliations:** Department of Biology, Integrated Sciences Complex, University of Massachusetts Boston, Boston, Massachusetts, United States of America; Universite de Fribourg, SWITZERLAND

## Abstract

Color vision in *Drosophila melanogaster* is based on the expression of five different color-sensing Rhodopsin proteins in distinct subtypes of photoreceptor neurons. Promoter regions of less than 300 base pairs are sufficient to reproduce the unique, photoreceptor subtype-specific *rhodopsin* expression patterns. The underlying *cis*-regulatory logic remains poorly understood, but it has been proposed that the *rhodopsin* promoters have a bipartite structure: the distal promoter region directs the highly restricted expression in a specific photoreceptor subtype, while the proximal core promoter region provides general activation in all photoreceptors. Here, we investigate whether the *rhodopsin* promoters exhibit a strict specialization of their distal (subtype specificity) and proximal (general activation) promoter regions, or if both promoter regions contribute to generating the photoreceptor subtype-specific expression pattern. To distinguish between these two models, we analyze the expression patterns of a set of hybrid promoters that combine the distal promoter region of one *rhodopsin* with the proximal core promoter region of another *rhodopsin*. We find that the function of the proximal core promoter regions extends beyond providing general activation: these regions play a previously underappreciated role in generating the non-overlapping expression patterns of the different *rhodopsins*. Therefore, *cis*-regulatory motifs in both the distal and the proximal core promoter regions recruit transcription factors that generate the unique *rhodopsin* patterns in a combinatorial manner. We compare this combinatorial regulatory logic to the regulatory logic of olfactory receptor genes and discuss potential implications for the evolution of *rhodopsins*.

## Author summary

Each type of sensory receptor neuron in our body expresses a specific sensory receptor protein, which allows us to detect and discriminate a variety of environmental stimuli. The regulatory logic that controls this spatially precise and highly restricted expression of sensory receptor proteins remains poorly understood. As a model system, we study the mechanisms that control the expression of different color-sensing Rhodopsin proteins in distinct subtypes of *Drosophila* photoreceptors, which is the basis for color vision. Compact promoter regions of less than 300 base pairs are sufficient to reproduce the non-overlapping *rhodopsin* patterns. However, the regulatory logic that underlies the combination (sometimes called ‘grammar’) of the *cis*-regulatory motifs (sometimes called ‘vocabulary’) within the *rhodopsin* promoters remains poorly understood. Here, we find that specific combinations of *cis*-regulatory motifs in the distal and the proximal core promoter regions of each *rhodopsin* direct its unique expression pattern.

## Introduction

**Fig 1:**
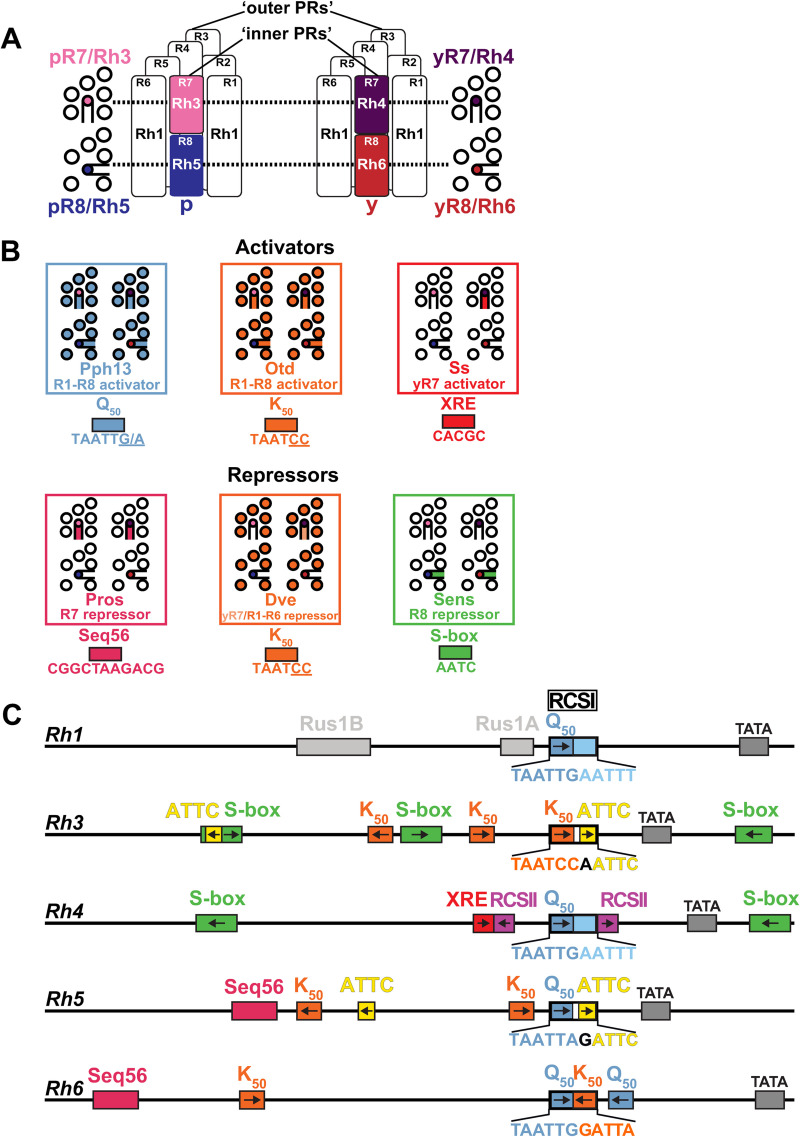
*Rhodopsin* expression in different photoreceptor subtypes. (A) Rhodopsins (Rhs) with different wavelength sensitivities are expressed in different photoreceptor (PR) subtypes. Schematic side views (center) show that ‘outer’ PRs R1-R6 (white) express Rh1, while ‘inner PRs’ R7/R8 (colored) express one of four different Rhs: pR7s express Rh3, yR7s express Rh4, pR8s express Rh5, and yR8s express Rh6. Flanking cross-sections show the different ‘inner PR’ subtypes at the level of R7s (pR7/Rh3 and yR7/Rh4, top) and R8s (pR8/Rh5 and yR8/Rh6, bottom). (B) Schematics representing the expression patterns of sequence-specific transcriptional activators (top) and repressors (bottom) that control *Rh* expression through specific motifs (colored boxes) that are also present in the *Rh* promoters in (C). (C) Schematics of the promoters of the five different *Rhs* that are expressed in the *Drosophila* eye and important *cis*-regulatory motifs (colored boxes, arrows indicate motif orientation). Each *Rh* promoter has a specific variant of the *Rhodopsin* Core Sequence I (RCSI) in its proximal core region. Colors highlight different sub-motifs that are explained in (B). *Rh4* has a unique XRE motif, while there are multiple motifs that are shared among other *Rhs* (e.g. K_50_, orange). See text for details.

A prerequisite for color vision is the expression of different wavelength-sensitive visual pigments in specific subtypes of photoreceptor neurons [1]. For instance, human rod photoreceptors (PRs) express Rhodopsin (Rh) that mediates vision at low light levels, while three different subtypes of cone PRs express one of three cone opsins that mediate color vision [2]. Similarly, the rod-equivalent *Drosophila* ‘outer’ PR class (R1-R6) expresses blue-green sensitive Rh1 and mediates dim light vision, while the cone-equivalent ‘inner’ PR class (R7/R8) mediates color vision (Fig 1A) [3,4]. Based on their *Rh* expression, the R7 and R8 PR types can be further subdivided into two subtypes, ‘p’ and ‘y’ (Fig 1A): pR7s express short UV-sensitive Rh3 and yR7s express long UV-sensitive Rh4, while pR8s express blue-sensitive Rh5 and yR8s express green-sensitive Rh6 (Fig 1A, right). This spatially precise expression of Rhs in specific PR neuron subtypes determines their wavelength sensitivity and generates PR neuron diversity.

Sequence-specific transcription factors have been identified that activate or repress *Rhs* through specific *cis*-regulatory motifs (Fig 1B and 1C) [4]. The two homeodomain transcription factors Pph13 [5,6] and Orthodenticle (Otd) [7,8] are expressed in all PRs and mediate broad activation of *Rhs* through Q_50_ (TAATTG/A) and K_50_ motifs (TAATCC), respectively (Fig 1B). The preferred binding to a Q_50_ or a K_50_ motif, respectively, is determined by the residue at position 50 of their homeodomain, either a glutamine (Q) or a lysine (K), which specifies DNA binding [9]. In contrast to the broadly expressed activators Pph13 and Otd, the PAS/bHLH transcription factor Spineless (Ss) is specifically expressed in the yR7/Rh4 PR subset (Fig 1B), where it activates *Rh4* through an XRE motif [10,11] (Fig 1C). Repressors restrict each *Rh* to a different PR subtype: Defective proventriculus (Dve), another K_50_-type homeodomain transcription factor, is expressed at low levels in the yR7/Rh4 subset and at high levels in R1-R6 PRs (Fig 1B), where it represses *Rh3*, *Rh5*, and *Rh6* through K_50_ motifs [12] (Fig 1C). The R7-specific homeodomain transcription factor Prospero (Pros) (Fig 1B) represses *Rh5* and *Rh6* in R7s through Seq56 motifs [13] (Fig 1C), while the R8-specific zinc-finger transcription factor Senseless (Sens) (Fig 1B) represses *Rh3* and *Rh4* in R8 PRs through S-box motifs [14] (Fig 1C). The transcription factor Runt has recently been identified as a novel regulator of R7 subtype specification that represses Rh4 fate, but it is unclear whether it represses *Rh4* directly [15].

Despite our knowledge of these key regulators, the motifs they bind to, and the fact that short promoter regions of less than 300 base pairs are sufficient to reproduce the spatial expression pattern of each *Rh* [8,16–18] (Fig 1C), the *cis*-regulatory logic that controls *Rh* expression in specific PR subtypes remains poorly understood. A pioneering study [18] proposed that the *Rh* promoters can be divided into two functionally specialized regions: the distal promoter region restricts the expression to a specific PR subtype and the proximal core promoter region provides general activation in all PRs. The boundary between the distal and proximal promoter region in this ‘bipartite promoter’ model is defined by the *Rhodopsin* Core Sequence I (RCSI) (Fig 1C) [18], which occurs in a very similar position right upstream of the TATA box in the proximal core region of all *Rh* promoters (Fig 1C). Consistent with the model’s proposed involvement of the proximal region in general activation [18], the RCSI is bound by the broadly expressed activators Otd and Pph13 [6]. Moreover, *cis*-regulatory motifs for activators and repressors that control *Rh* subtype specificity have indeed been identified in the distal promoter region [8,13]. However, the ‘bipartite promoter’ model has been proposed before *Rh5* and *Rh6* were cloned. It is therefore unclear how absolute the suggested distal-proximal specialization is and whether it applies to all *Rhs*. Moreover, an R8 repressor motif (S-box) has more recently been identified [14] in the proximal core promoters of *Rh3* and *Rh4* (Fig 1C) and our previous work revealed that the RCSI motifs of different *Rhs* contain conserved repressor motifs (S1 and S2 Figs) that are critical for subtype-specific expression [19].

**Fig 2:**
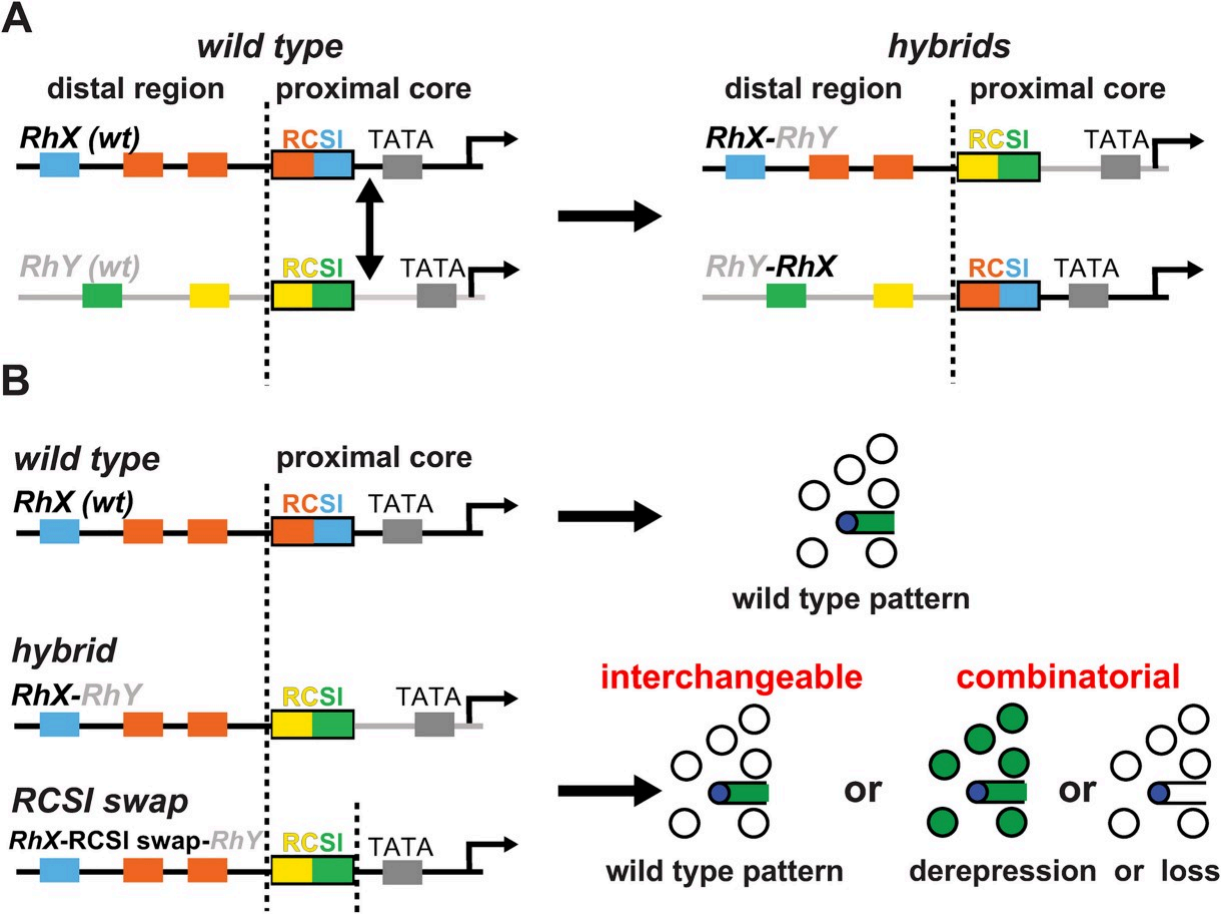
*Rhodopsin* promoter hybrids and motif swaps to distinguish between two models for proximal core promoter function. (A) Left: Schematic of two hypothetical wild type (*wt*) *Rh* promoters (*RhX* and *RhY*) with their distal region and their proximal core region; the latter includes the RCSI motif. The boundary between the distal and proximal core promoter regions just 5’ of the RCSI motif is indicated by a vertical dashed line; replacements of the proximal core promoter regions (indicated by double arrow) were made at this breakpoint. Right: The two resulting hybrid promoters, *RhX*-*RhY* and *RhY*-*RhX* (the promoter that provided the distal region is named first), have novel distal and proximal motif combinations (colored boxes). (B) Experimental logic. A wild type *Rh* promoter drives reporter expression (green) in a specific photoreceptor subset (top). If the replacement of the entire proximal core promoter (middle) or only the RCSI motif (bottom) with the one from another *Rh* promoter resulted in the same wild type expression pattern, then these regions are ‘interchangeable’ between two *Rhs*. They would thus follow the ‘interchangeable core’ model (bottom right). Alternatively, if such replacements resulted in different patterns—derepression or lack of expression—this means that important proximal motifs have been lost. The latter outcome would suggest that generating the correct pattern requires specific distal and proximal motif combinations, as proposed by the ‘combinatorial core’ model (bottom right).

Here, we revisit the ‘bipartite promoter’ model that predicts that the proximal core promoters of different *Rhs* are interchangeable because they are specialized in providing broad activation in all PRs. For simplicity, we renamed it ‘interchangeable core’ model to distinguish it from the alternative ‘combinatorial core’ model that proposes that the proximal region contributes to subtype-specific Rh expression in a combinatorial manner (Fig 2A and 2B). To distinguish between these two models, we generated a set of hybrid *Rh* promoters that fuse the distal region of one *Rh* to the proximal core region of another *Rh* (Fig 2A, right). We also swapped the RCSI motif between different Rhs (Fig 2B) to compare the effects of replacing this specific proximal motif with the effects of replacing the entire proximal region. Our data strongly support the ‘combinatorial core’ model, i.e. that the distal and proximal *Rh* promoter motifs are matched to generate a unique *Rh* expression pattern. The data also provide further evidence for a key role of the RCSI motif in generating restricted expression patterns. We compare this combinatorial logic to the regulatory logic of olfactory receptor genes and discuss potential implications for the evolution of *Rhs*.

## Results

To determine whether the *Rh* regulation follows the ‘interchangeable core’ model (interchangeable proximal core, only distal motifs generate subtype specificity) or the alternative ‘combinatorial core’ model (a combination of specific distal and proximal motifs generates subtype specificity), we generated two types of constructs (Fig 2A and 2B). First, we fused the distal promoter region of one *Rh* with the proximal core promoter region of another *Rh* (Fig 2A). The consistent break/fusion point, where the distal and proximal regions were separated and recombined, was the first base pair upstream of the RCSI motif (dashed vertical lines in Fig 2A and 2B). We used the following nomenclature for the hybrids: The *Rh* that provided the distal promoter region (e.g. *Rh3*) is named first and is followed by the *Rh* that provided the proximal core region (e.g. *Rh4*), e.g. ‘*Rh3*-*Rh4* hybrid’. Using this hybrid promoter approach, we surprisingly found examples for both the ‘interchangeable core’ model as well as the ‘combinatorial core’ model (see below and Discussion).

Second, we specifically swapped the unique RCSI variants of the different *Rhs* to investigate the role of the proximal RCSI motifs in generating the spatial pattern (Fig 2B, bottom left) independently of the downstream core promoter region. The *Rh* that provided the promoter context for the RCSI swap is named first (e.g. *Rh3*), followed by ‘RCSI swap’ and the *Rh* that provided the RCSI motif for the swap (e.g. *Rh4*), e.g. ‘*Rh3*-RCSI swap*-Rh4*’. To facilitate comparisons, we inserted all wild type, hybrid, and RCSI swap constructs in the same landing site (see Materials and methods).

### Special features of the *Rh4* promoter and compatibility of its distal region with the proximal core of *Rh3* and *Rh5*

The bipartite *Rh* promoter model [18] was largely based on the mutational analysis of the promoters of *Rh3* and *Rh4* that are expressed in two different R7 subsets [18]; *Rh5* and *Rh6* had not been cloned at the time. Therefore, we first compared the *Rh3* and *Rh4* promoter signatures with the ones of *Rh5* and *Rh6* (Figs 1C, S1 and S2). The *Rh4* promoter differs from the other *Rhs* in several unique features. It contains a highly conserved distal XRE motif that mediates subtype-specific activation through the yR7-specific transcription factor Ss (Fig 1B) [11]. In contrast, *Rh3*, *Rh5*, and *Rh6* share distal K_50_ motifs (S1B Fig) for the broad activator Otd that is expressed in all PRs (Fig 1B) [8]. Second, the proximal core of the *Rh4* promoter has a rather generic RCSI activator motif (TAATTGAATTT; Fig 1C), which lacks the repressor sub-motifs within the *Rh3*, *Rh5*, and *Rh6* RCSIs (colored boxes and areas within RCSI motifs in Figs 1C, S1A–S1D, and S2A–S2D) [19]. *Rh4* has only a single known type of repressor motif, a distal and a proximal S-box for R8 repression through Sens [14], while *Rh3*, *Rh5*, and *Rh6* have several repressor motifs, such as K_50_/Dve motifs or ATTC/y repressor motifs that occur in the distal promoter and within their RCSI (S2B–S2D Fig).

**Fig 3:**
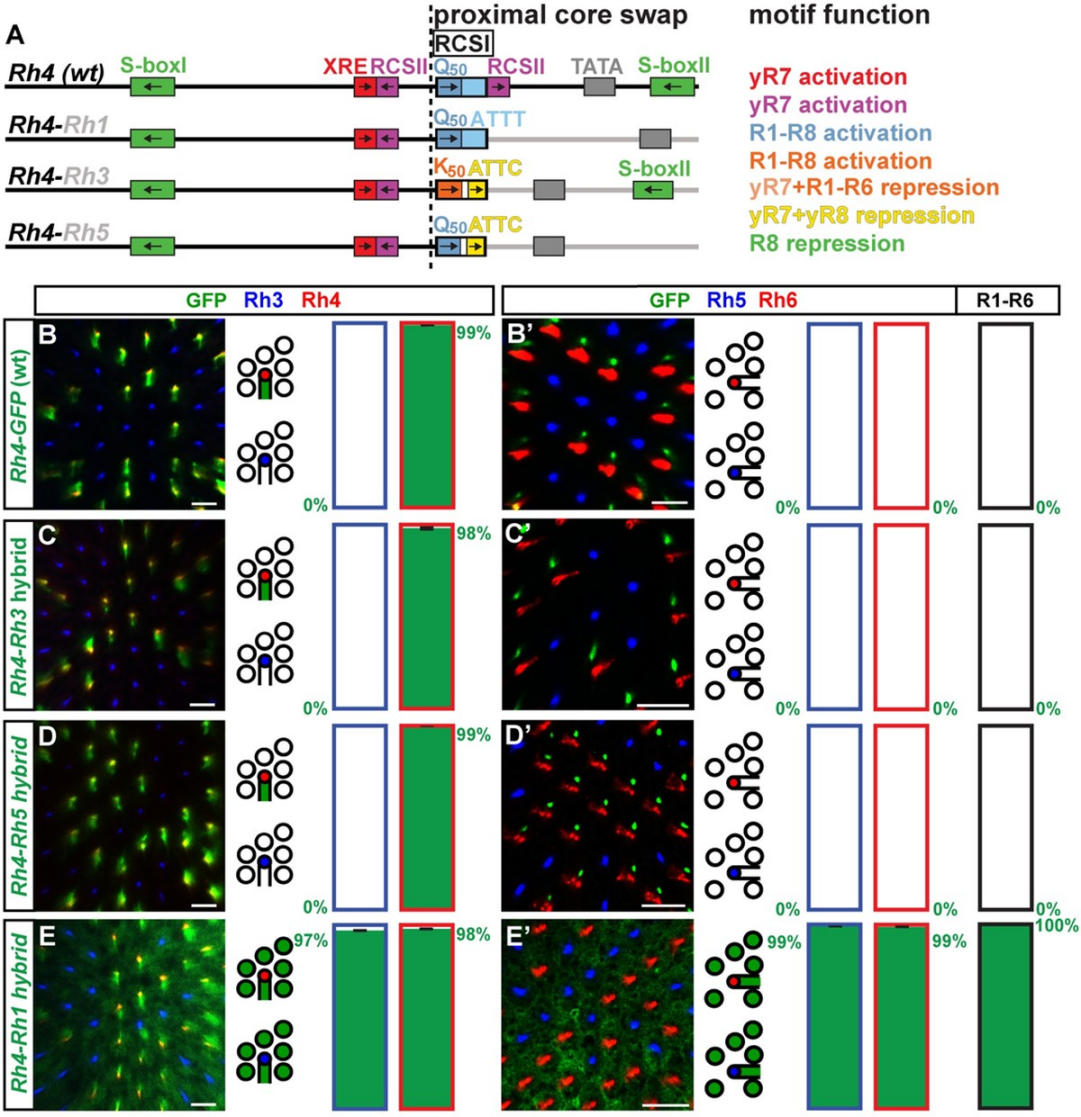
Compatibility of the unique *Rh4* motifs with the proximal core motifs of *Rh3* and *Rh5*. (A) Schematic comparison of the wild type (*wt*) *Rh4* promoter and hybrids of the distal *Rh4* promoter combined with the proximal core promoters of *Rh1*, *Rh3*, or *Rh5*. Note that the RCSI motifs of *Rh1* and *Rh4* are identical and that *Rh3* and *Rh4* share a proximal S-box motif for R8 repression. The dotted vertical line indicates the break/fusion point of the hybrids immediately upstream of the respective RCSI motif that is found in a similar position in all *Rhs*. (B)—(E) Wild type *Rh4 vs*. hybrid promoters driving GFP reporter expression (green) in the R7 layer. Rh3 (blue) labels pR7s and Rh4 (red) labels yR7s. Bar graphs show GFP co-expression in the Rh3 or Rh4 subset, respectively. (B’)—(E’) Wild type *Rh4 vs*. hybrid promoters driving GFP reporter expression (green) in the R8 layer. Rh5 (blue) labels pR8s and Rh6 (red) labels yR8s. Bar graphs show GFP co-expression in the Rh5, Rh6, or R1-R6 subset. Green numbers indicate the mean percentage of GFP co-expressing photoreceptors, error bar represents standard error of the mean. (B) and (B’) The wild type *Rh4* promoter drives subtype-specific GFP expression in the yR7/Rh4 subset. Green staining in (B’) is from the axons of yR7s that traverse the R8 layer. N = 9 retinas and n = 949 R7s for (B); N = 9 retinas, n = 869 R8s and 5,214 R1-R6 PRs for (B’). (C) and (C’) The *Rh4*-*Rh3* hybrid drives subtype-specific GFP reporter expression in yR7s. Green staining in (C’) is from the axons of yR7s that traverse the R8 layer. N = 11 retinas and n = 1,327 R7s for (C); N = 12 retinas, n = 1,192 R8s and 7,152 R1-R6 PRs for (C’). (D) and (D’) The *Rh4*-*Rh5* hybrid drives subtype-specific GFP expression in yR7s. Green staining in (D’) is from the axons of yR7s that traverse the R8 layer. N = 12 retinas and n = 1,793 R7s for (D); N = 10 retinas, n = 730 R8s and 4,380 R1-R6 PRs for (D’). (E) and (E’) The *Rh4*-*Rh1* hybrid drives broad GFP expression in both R7 and R8 photoreceptor subtypes as well as R1-R6. N = 12 retinas and n = 1,024 R7s for (E); N = 10 retinas, n = 589 R8s and 3,534 R1-R6 PRs for (E’). Scale bars, 10 μm.

Since the distal *Rh4* promoter already provides yR7 subtype-restricted activation through XRE/Ss and the motif analysis suggests that it requires minimal repression, we hypothesized that hybrids with a distal *Rh4* promoter (Fig 3A) would be the most likely ones to follow the ‘interchangeable core’ model and be compatible with the proximal core regions of other *Rhs*. Indeed, like a wild type *Rh4* promoter (Fig 3B and 3B’), the ***Rh4*-*Rh3*** (Fig 3C and 3C’) and ***Rh4*-*Rh5*** hybrids (Fig 3D and 3D’) drove subtype-specific expression in the yR7/Rh4 subset. There was no significant difference between the hybrid expression patterns and the one of the wild type *Rh4* promoter (p>0.4 and p>0.06; Mann-Whitney U-test) and the two hybrids thus followed the ‘interchangeable core’ model. Moreover, the *Rh4-Rh3* and *Rh4-Rh5* hybrids demonstrate that the distal XRE/Ss and RCSII motifs can provide combinatorial activation with other proximal motifs in addition to the *Rh4* RCSI and RCSII. This distal-proximal compatibility does not automatically mean that the RCSI motifs were interchangeable, because specifically swapping the *Rh4* RCSI with the *Rh3* RCSI in the *Rh4* promoter (*Rh4*-RCSI swap-*Rh3*, S3A Fig) caused faint derepression in a substantial fraction of pR7s (S3B and S3B’ Fig); motifs or features downstream of the *Rh3* RCSI thus prevented this expansion into the pR7 PR subtype in the hybrid. Taken together, in agreement with the ‘interchangeable core’ model, the replacement of *Rh4*’s proximal core promoter with the one of *Rh3* or *Rh5* did not significantly affect the subtype-specific expression pattern.

### The distal *Rh4* promoter region is incompatible with the proximal core of *Rh1* and *Rh6*

In contrast to the *Rh4*-*Rh3* and *Rh4*-*Rh5* hybrids that drove *Rh4*-like PR subtype-specific patterns, the *Rh4*-*Rh1* and the *Rh4*-*Rh6* hybrid drove novel, broader expression patterns, which is at odds with the ‘interchangeable core’ model. The ***Rh4*-*Rh1*** hybrid drove broad expression in all PRs (Fig 3E and 3E’), despite retaining the RCSI motif that is the same in *Rh4* and *Rh1* (Fig 1C). The broad expression and expansion into all PR subsets were thus caused by certain motifs or features downstream of the *Rh1* RCSI that are still unknown.

**Fig 4:**
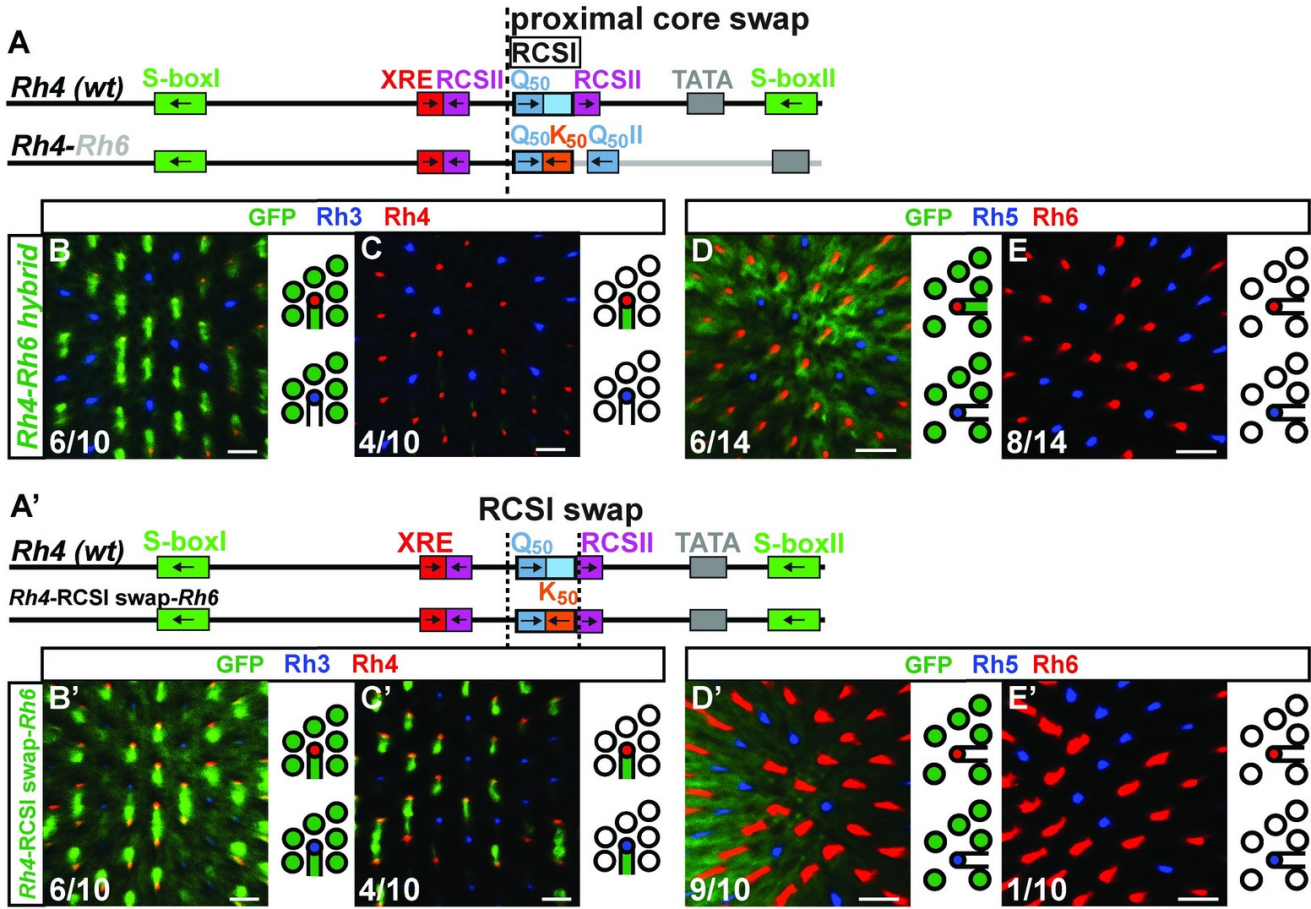
Incompatibility of distal *Rh4* and proximal *Rh6*. (A) Schematic comparison of the wild type *Rh4* promoter and the *Rh4-Rh6* hybrid. The dotted vertical line indicates the break/fusion point of the hybrid immediately upstream of the RCSI motif that is found in a similar position in all *Rhs*. (B)—(C) and (B’)—(C’) Hybrid and RCSI swap driving GFP reporter expression (green) in the R7 layer. Rh3 (blue) labels pR7s and Rh4 (red) labels yR7s. (D)—(E) and (D’)—(E’) Hybrid and RCSI swap driving GFP reporter expression (green) in the R8 layer. Rh5 (blue) labels pR8s and Rh6 (red) labels yR8s. (B) and (C) The *Rh4-Rh6* hybrid drives variable reporter expression. 6/10 retinas exhibit strong GFP expression in yR7s and R1-R6, 4/10 retinas show weak GFP expression only in yR7s. N = 10 retinas and n = 841 R7s. (D) and (E) The *Rh4-Rh6* hybrid drives variable reporter expression. 6/14 retinas show strong GFP expression in yR8s and R1-R6, 8/14 retinas show no detectable GFP expression in R8s or R1-R6. N = 14 retinas, n = 902 R8s and 5,412 R1-R6 PRs. (A’) Schematic comparison of the wild type *Rh4* promoter and the specific RCSI swap with the *Rh6* RCSI (indicated by the dotted vertical lines). Note the shared Q_50_ motif in the RCSI. (B’) and (C’) The swap of the *Rh4* RCSI with the *Rh6* RCSI in the *Rh4* promoter context drives variable GFP reporter expression. 6/10 retinas show strong GFP expression in both R7 subtypes and R1-R6, 4/10 retinas show GFP expression in both R7 subtypes but not R1-R6. N = 10 retinas and n = 646 R7s. (D’) and (E’) The swap of the *Rh4* RCSI with the *Rh6* RCSI in the *Rh4* promoter context drives variable GFP reporter expression. 9/10 retinas show strong GFP expression in R1-R6, 1/10 retinas show no detectable GFP expression in R8 or R1-R6. N = 10 retinas, n = 642 R8s and 3,852 R1-R6 PRs. Scale bars, 10 μm.

The ***Rh4*-*Rh6*** hybrid (Fig 4A) drove variable, broadened expression that included the R1-R6 subset, the yR7/Rh4 subset, and the yR8/Rh6 subset (Fig 4B–4E). The replacement of the RCSI motif played a role in the expansion into other PR subtypes, because specifically swapping the *Rh4* RCSI with the *Rh6* RCSI in the *Rh4* promoter context (*Rh4*-RCSI swap-*Rh6*, Fig 4A’) resulted in a similarly variable expression in R1-R6 PRs, but also in the pR7/Rh3 subset and not in R8s (Fig 4B’–4E’). Moreover, it is likely that motifs or features downstream of the *Rh6* RCSI were involved in the broader expression, for instance the second Q_50_/Pph13 activator motif.

Summarizing the results for the hybrids with the distal *Rh4* promoter region, two (*Rh4*-*Rh3* and *Rh4*-*Rh5*) generated a *Rh4*-like pattern (despite conflicting activator and repressor motifs, see S1 Text) and thus revealed alternative proximal core combinations and a motif flexibility that is consistent with the ‘interchangeable core’ model. Conversely, the two other hybrids (*Rh4*-*Rh1* and *Rh4*-*Rh6*) yielded novel, broader patterns which is consistent with the ‘combinatorial core’ model because their distal and proximal regions were not compatible with subtype-restricted expression.

### Hybrid promoters reveal alternative motif combinations for expression in the same photoreceptor subtype

**Fig 5:**
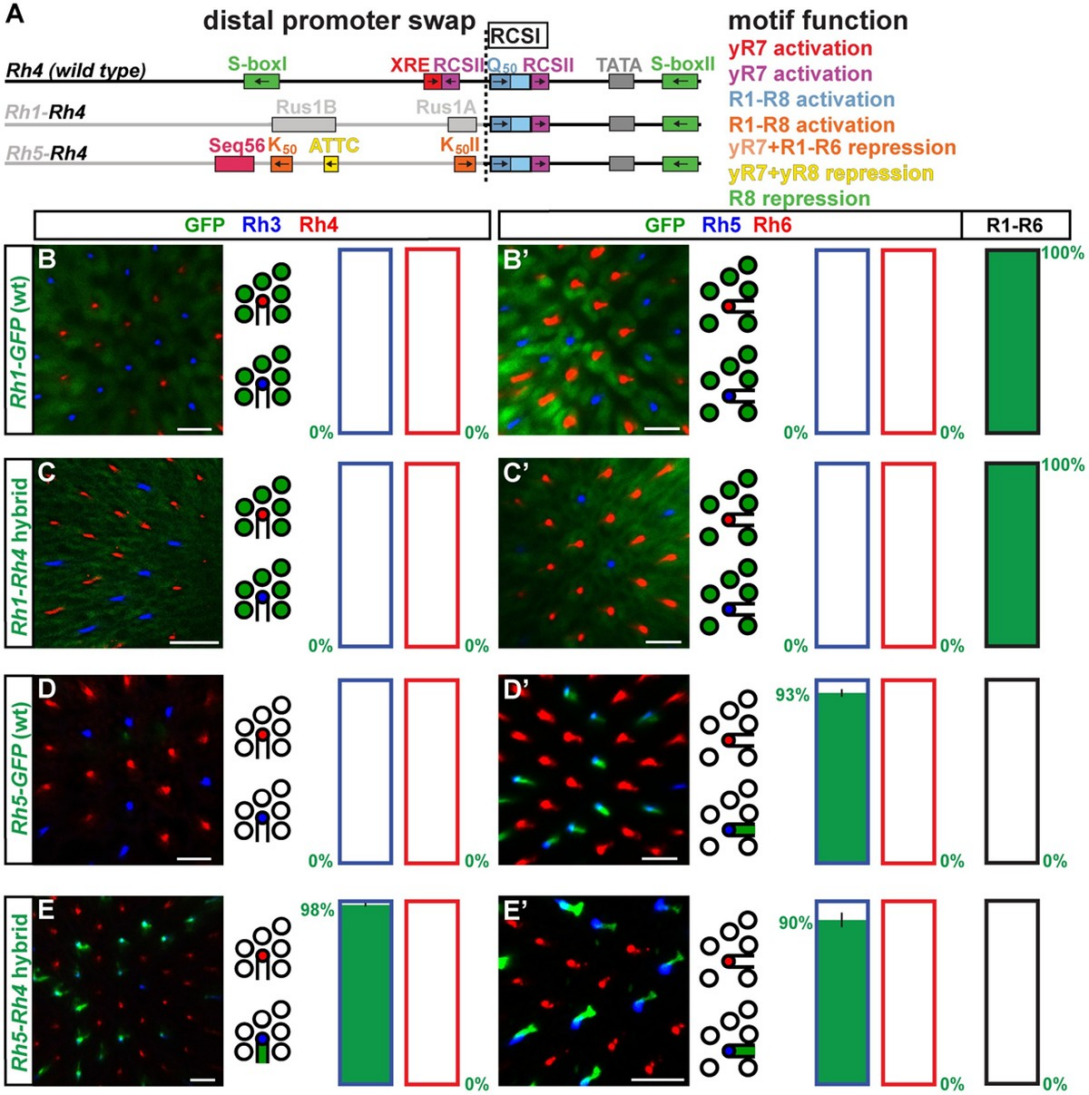
Hybrid promoters reveal compatible and incompatible distal and proximal motif combinations. (A) Schematic comparison of wild type *Rh4* promoter and hybrids of the proximal core *Rh4* promoter combined with the distal promoters of *Rh1* or *Rh5*. Note that *Rh4* shares no distal motifs with *Rh1* or *Rh5*. The dotted vertical line indicates the break/fusion point of the hybrids. (B)—(E) Wild type *vs*. hybrid promoters driving GFP reporter expression (green) in the R7 layer. Rh3 (blue) labels pR7s and Rh4 (red) labels yR7s. Bar graphs show GFP co-expression in the Rh3 or Rh4 subset, respectively. (B’)—(E’) Wild type *vs*. hybrid promoters driving GFP reporter expression (green) in the R8 layer. Rh5 (blue) labels pR8s and Rh6 (red) labels yR8s. Bar graphs show GFP co-expression in the Rh5, Rh6, or R1-R6 subset. Green numbers indicate the mean percentage of GFP co-expressing photoreceptors, error bar represents standard error of the mean. (B) and (B’) The wild type *Rh1* promoter drives subtype-specific GFP expression in the R1-R6 subset. N = 9 retinas and n = 630 R7s for (B); N = 8 retinas, n = 560 R8s and 3,360 R1-R6 PRs for (B’). (C) and (C’) The *Rh1*-*Rh4* hybrid drives subtype-specific GFP reporter expression in R1-R6 photoreceptors. N = 13 retinas and n = 1,014 R7s for (C); N = 8 retinas, n = 512 R8s and 3,072 R1-R6 PRs for (C’). (D) and (D’) The wild type *Rh5* promoter drives subtype-specific GFP expression in the yR8/Rh5 subset. N = 10 retinas and n = 767 R7s for (D); N = 11 retinas, n = 997 R8s and 5,982 R1-R6 PRs for (D’). (E) and (E’) The *Rh5*-*Rh4* hybrid drives GFP expression in the pR7 subtype and the pR8 subtype. N = 11 retinas and n = 1,894 R7s for (E); N = 9 retinas, n = 566 R8s and 3,396 R1-R6 PRs for (E’). Scale bars, 10 μm.

In a complementary approach to analyze how the proximal core promoter region and a specific distal promoter region generate a spatially restricted *Rh* pattern, we kept *Rh4*’s proximal core promoter region constant and combined it with the distal promoter regions of other *Rhs* (Figs 5A and S4A).

Like a wild type *Rh1* promoter, the ***Rh1*-*Rh4*** hybrid drove subset-specific expression in R1-R6 (compare Fig 5B and 5B’ to Fig 5C and 5C’; p>0.9, Mann-Whitney U-test). The hybrid retained the *Rh1* RCSI, which is identical with *Rh4* (Fig 1C). The replacement of the downstream region with the corresponding proximal *Rh4* sequences (Fig 5A) did not affect expression in the R1-R6/Rh1-expressing subset.

The ***Rh5*-*Rh4*** hybrid drove expression in the pR8/Rh5 subset, where *Rh5* is expressed (Fig 5D and 5D’), but additionally in the pR7/Rh3 subset (Fig 5E and 5E’), in which neither of the two contributing wild type *Rhs* promoters drive expression. This hybrid thus generated a novel expression pattern that labeled both ‘pale’ subtypes (pR7 and pR8).

The ***Rh6*-*Rh4*** hybrid (S4A Fig) did not drive detectable expression in PRs (S4B–S4E Fig) and specifically swapping the *Rh6* RCSI with the *Rh4* RCSI (S4A’ Fig) gave a similar result (S4B’–S4E’ Fig), which means that the distal and proximal promoter regions of *Rh6* and *Rh4* as well as their RCSI motifs were not interchangeable.

**Fig 6:**
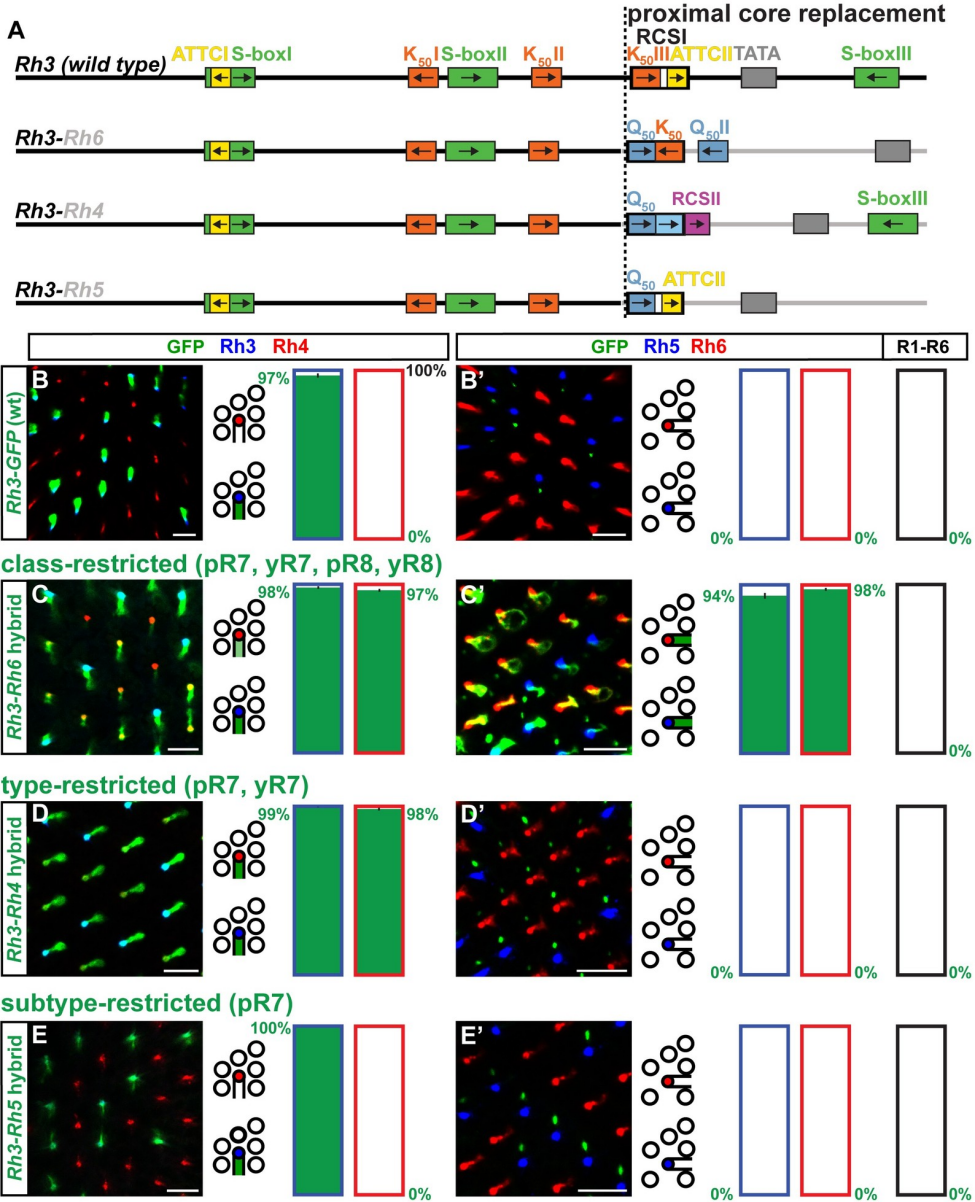
Novel motif combinations yield a series of progressively restricted expression patterns. (A) Schematic comparison of wild type *Rh3* promoter and hybrids of the distal *Rh3* promoter combined with the proximal core promoters of *Rh4*, *Rh5*, or *Rh6*. Note in the proximal region the shared K_50_ motif within the *Rh6* RCSI and the ATTCII motif at the same position within the *Rh5* RCSI, as well as the same spacing of the *Rh5* RCSI and the TATA box. The dotted vertical line indicates the break/fusion point of the hybrids. (B)—(E) Wild type *vs*. hybrid promoters driving GFP reporter expression (green) in the R7 layer. Rh3 (blue) labels pR7s and Rh4 (red) labels yR7s. Bar graphs show GFP co-expression in the Rh3 or Rh4 subset, respectively. (B’)—(E’) Wild type reporter *vs*. hybrid promoter driving GFP reporter expression (green) in the R8 layer. Rh5 (blue) labels pR8s and Rh6 (red) labels yR8s. Bar graphs show GFP co-expression in the Rh5, Rh6, or R1-R6 subset. Green numbers indicate the mean percentage of GFP co-expressing photoreceptors, error bar represents standard error of the mean. (B) and (B’) The wild type *Rh3* promoter drives subtype-specific GFP expression in the pR7/Rh3 subset. Green staining in (B’) is from the axons of pR7s that traverse the R8 layer. N = 18 retinas and n = 1,188 R7s for (B); N = 11 retinas, n = 958 R8s and 5,748 R1-R6 PRs for (B’). (C) and (C’) The *Rh3*-*Rh6* hybrid drives photoreceptor class-restricted GFP expression in all ‘inner’ photoreceptors, i.e. both the R7 and R8 photoreceptor types (pR7s, yR7s, pR8s, and yR8s). N = 10 retinas and n = 1,307 R7s for (C); N = 12 retinas, n = 872 R8s and 5,232 R1-R6 PRs for (C’). (D) and (D’) The *Rh3*-*Rh4* hybrid drives photoreceptor type-restricted GFP expression in both R7 subtypes (pR7s and yR7s). GFP signal in (D’) is from traversing R7 axons. N = 12 retinas and n = 1,569 R7s for (D); N = 11 retinas, n = 708 R8s and 4,248 R1-R6 PRs for (D’). (E) and (E’) The *Rh3*-*Rh5* hybrid drives photoreceptor subtype-specific GFP expression in pR7s. GFP signal in (D’) is from pR7 axons. N = 11 retinas and n = 1,133 R7s for (E); N = 9 retinas, n = 967 R8s and 5,802 R1-R6 PRs for (E’). Scale bars, 10 μm.

The ***Rh3*-*Rh4*** hybrid (Fig 6A) drove expression in the pR7/Rh3 subset, where *Rh3* is expressed (Fig 6B and 6B’), but also the yR7/Rh4 subset (Fig 6D and 6D’) [18], which resembles an addition of the expression patterns of both *Rhs* that contributed their promoter regions to the hybrid.

Summarizing the results for the hybrids with a constant proximal *Rh4* promoter region, only the *Rh1*-*Rh4* hybrid generated a subtype-restricted expression pattern that resembled the one *Rh1* that provided the distal promoter region. Of the other hybrid combinations, one (*Rh6*-*Rh4*) did not drive any pattern while two (*Rh3*-*Rh4*, and *Rh5*-*Rh4*) generated novel patterns that involved more than one PR subtype. These three hybrids are thus consistent with the ‘combinatorial core’ model.

### Novel distal and proximal motif combinations yield a series of progressively restricted expression patterns

Since the *Rh3*, *Rh5*, and *Rh6* promoters share high-affinity K_50_ and Q_50_ homeodomain motifs, whose arrangements and orientations are evolutionarily conserved (S2 Fig and S1 Text), we next asked whether these similarities are an indicator for the compatibility of the respective distal and proximal promoter regions (Fig 6A). In contrast to the pR7 subtype-restricted pattern of the wild type *Rh3* promoter (Fig 6B and 6B’), the ***Rh3*-*Rh6*** hybrid drove a novel, ‘pan-inner’ PR expression pattern in both R7 subtypes and both R8 subtypes (Fig 6C and 6C’). The *Rh3*-*Rh6* hybrid therefore labeled all four subtypes of the ‘inner PR’ class: pR7/Rh3, yR7/Rh4, pR8/Rh5, and yR8/Rh6.

The ***Rh3*-*Rh4*** hybrid (also see above) drove another novel expression pattern in both the pR7/Rh3 subset as well as the yR7/Rh4 subset. This ‘pan-R7’ pattern was more restricted than the ‘pan inner’ pattern of *Rh3*-*Rh6* since it specifically labeled the subtypes of the R7 PR type, pR7/Rh3 and yR7/Rh4 (Fig 6D and 6D’) [18]. Therefore, the expression pattern of the *Rh3*-*Rh4* hybrid resembled the addition of the expression patterns of both contributing *Rhs*.

Like a wild type *Rh3* promoter, the ***Rh3*-*Rh5*** hybrid drove highly restricted, subtype-specific reporter expression in the pR7/Rh3 subset (compare Fig 6B and 6B’ to 6E and 6E’; p>0.3, Mann-Whitney U test). The proximal promoter region of *Rh5* resembles the one of *Rh3* because it preserves the proximal ATTC/y repressor motif repeat as well as the spacing of the RCSI to the TATA box (Figs 6A, S2B and S2C). The proximal core promoter signatures of *Rh3* and *Rh5* were thus equivalent when paired with distal *Rh3* motifs.

Summarizing the data for the *Rh3*, *Rh5*, and *Rh6* promoters that share several high-affinity homeodomain motifs, the outcome of only one (*Rh3*-*Rh5*) of the three hybrids was consistent with the ‘interchangeable core’ model. In contrast, the *Rh3*-*Rh4* hybrid and the *Rh3*-*Rh6* hybrid generated novel patterns that labeled more than one ‘inner’ PR subtype; these two hybrids were thus consistent with the ‘combinatorial core’ model. Strikingly, the simple proximal core promoter swaps in these three hybrids generated a series of progressively restricted spatial expression patterns, i.e. class>type>subtype (see Discussion).

### The role of the proximal RCSI motif in generating the spatial pattern

**Fig 7:**
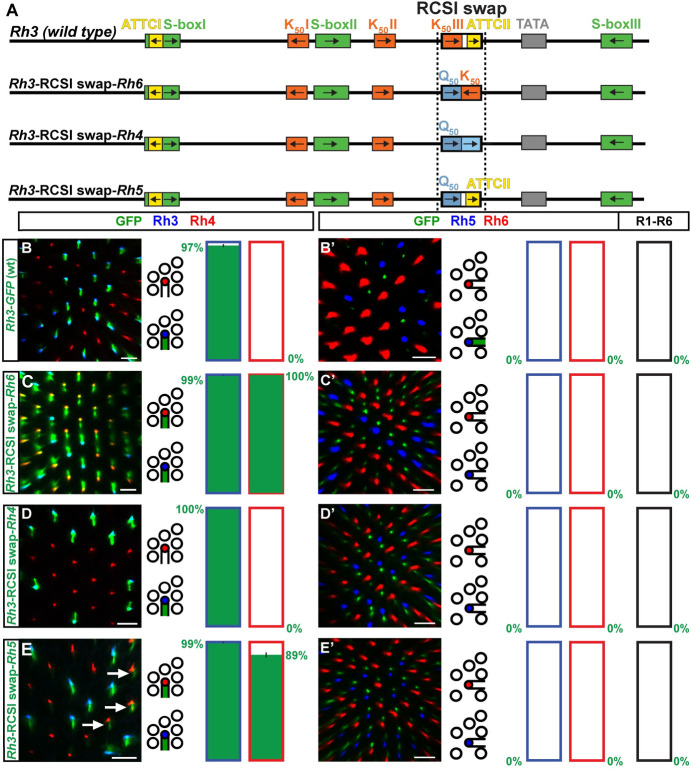
The role of the proximal RCSI motif in the generation of the spatial pattern. (A) Schematic comparison of the wild type *Rh3* promoter and specific swaps of the *Rh3* RCSI with the RCSI motif of *Rh4*, *Rh5*, or *Rh6*. The dotted vertical line indicates the swapped motif. (B)—(E) Wild type *Rh3* promoter *vs*. RCSI swaps driving GFP reporter expression (green) in the R7 layer. Rh3 (blue) labels pR7s and Rh4 (red) labels yR7s. Bar graphs show GFP co-expression in the Rh3 or Rh4 subset, respectively. (B’)—(E’) Wild type *Rh3* promoter *vs*. RCSI swaps driving GFP reporter expression (green) in the R8 layer. Rh5 (blue) labels pR8s and Rh6 (red) labels yR8s. Bar graphs show GFP co-expression (green) in the Rh5, Rh6, or R1-R6 subset. Green numbers indicate the mean percentage of co-expressing photoreceptors, error bar represents standard error of the mean. (B) and (B’) The wild type *Rh3* promoter drives subtype-specific GFP expression in the pR7/Rh3 subset. Green staining in (B’) is from pR7 photoreceptor axons that traverse the R8 layer. Statistics are same as in Fig 6B–6B’. (C) and (C’) The swap of the *Rh3* RCSI with the *Rh6* RCSI in the *Rh3* promoter context causes an expansion of GFP into the yR7/Rh4 subset. N = 12 retinas and n = 1,074 R7s for (C); N = 9 retinas, n = 691 R8s and 4,146 R1-R6 PRs for (C’). (D) and (D’) The swap of the *Rh3* RCSI with the *Rh4* RCSI in the *Rh3* promoter context has no obvious effect on subtype-specific expression in the pR7/Rh3 subset. N = 13 retinas and n = 987 R7s for (D); N = 10 retinas, n = 897 R8s and 5,382 R1-R6 PRs for (D’). (E) and (E’) The swap of the *Rh3* RCSI with the *Rh5* RCSI in the *Rh3* promoter context causes derepression of GFP in the yR7/Rh4 subset (white arrows). N = 10 retinas and n = 650 R7s for (E); N = 10 retinas, n = 877 R8s and 5,262 R1-R6 PRs for (E’). Scale bars, 10 μm.

After we identified hybrids in which the replacement of the proximal core promoter region—including the RCSI motif–was incompatible with subtype-specific expression, we asked how much of the spatial expansion could be explained by the specific swap of the RCSI motif (Fig 7A).

Similar to the *Rh3-Rh6* hybrid that generated a ‘pan-inner’ PR pattern that included both R7 and both R8 subtypes, the specific swap of the *Rh3* RCSI with the *Rh6* RCSI in the *Rh3* promoter context (Fig 7B and 7B’) caused expression in both R7 subsets (Fig 7C). However, the RCSI swap lacked R8 expression (Fig 7C’) [19,20]. The lack of expression in R8 is likely due to the preservation of the proximal S-box/R8 repressor motif for Sens (Fig 7A) in the RCSI swap experiment, while its replacement in the *Rh3-Rh6* hybrid would allow derepression in R8s [14].

**Fig 8:**
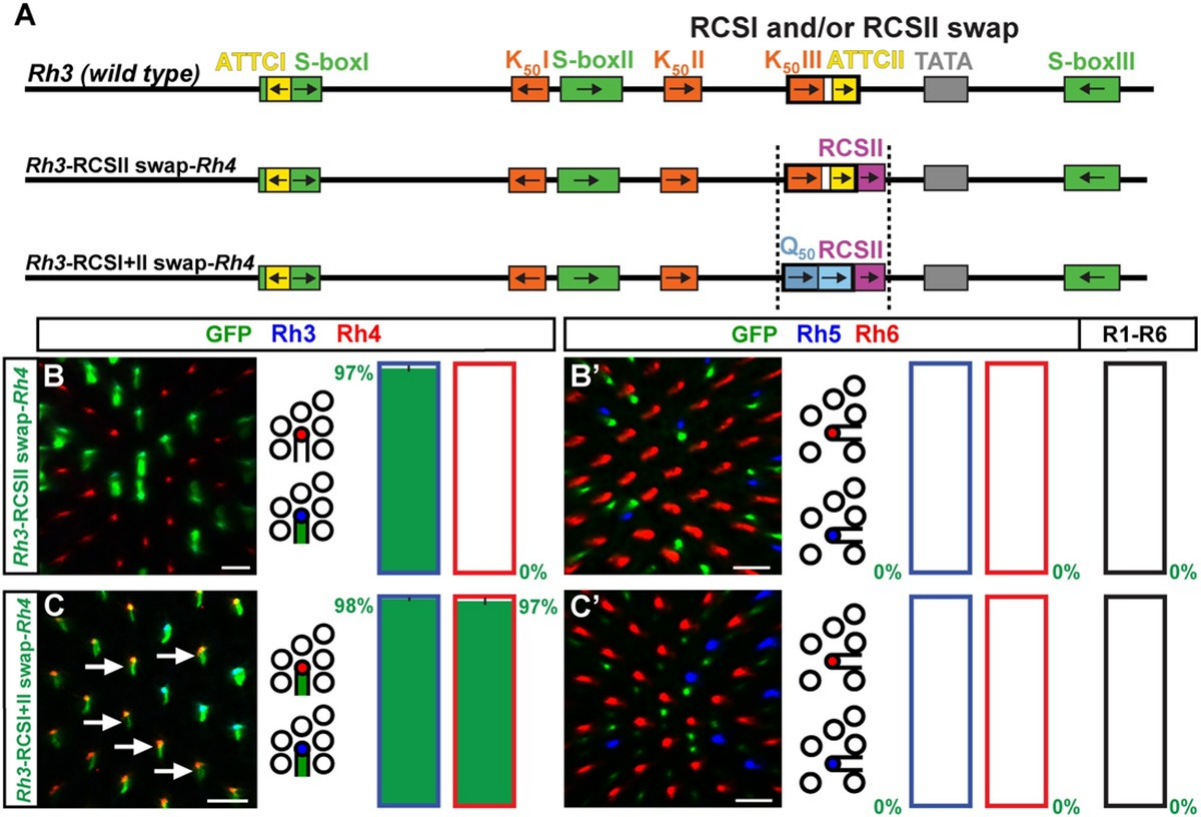
Incompatibility of the *Rh3* promoter with the *Rh4* RCSI and RCSII. (A) Wild type *Rh3* promoter (top) compared with addition of the *Rh4* RCSII (middle) or swap of the *Rh3* RCSI with the *Rh4* RCSI and *Rh4* RCSII (bottom, indicated by dotted vertical lines). Note that the *Rh3* and *Rh4* RCSIs do not share any motifs. (B) and (C) RCSI/II swap promoter driven GFP reporter expression (green) in the R7 layer. Rh3 (blue) labels pR7s and Rh4 (red) labels yR7s. Bar graphs show GFP co-expression in the Rh3 or Rh4 subset, respectively. Green numbers indicate the mean percentage of co-expressing photoreceptors, error bar represents standard error of the mean. (B’) and (C’) RCSI/II swap promoter driven GFP reporter expression (green) in the R8 layer. Rh5 (blue) labels pR8s and Rh6 (red) labels yR8s. Bar graphs show GFP co-expression (green) in the Rh5, Rh6, or R1-R6 subset. Green numbers indicate the mean percentage of co-expressing photoreceptors, error bar represents standard error of the mean. (B) and (B’) The addition of the *Rh4* RCSII to the *Rh3* promoter has no effect on subtype-specific expression of GFP in the pR7/Rh3 subset. N = 10 retinas and n = 692 R7s for (B); N = 11 retinas, n = 920 R8s and 5,520 R1-R6 PRs for (B’). (C) and (C’) The swap of the *Rh3* RCSI with both the *Rh4* RCSI and *Rh4* RCSII causes derepression in most yR7s (arrows). N = 10 retinas and n = 721 R7s for (C); N = 9 retinas, n = 871 R8s and 5,226 R1-R6 PRs for (C’). Scale bars, 10 μm.

In contrast to the pan-R7 pattern of the *Rh3-Rh4* hybrid, neither the specific swap of the *Rh3* RCSI with the *Rh4* RCSI (Fig 7D and 7D’) [18] nor specifically adding the *Rh4* RCSII without the *Rh4* RCSI (Fig 8A, 8B and 8B’) caused a pan-R7 pattern. However, the swap of the *Rh3* RCSI with both the *Rh4* RCSI and the neighboring *Rh4* RCSII caused the reporter expansion into yR7s (Fig 8C and 8C’).

The subtype-specific pattern of the *Rh3-Rh5* hybrid suggests that the RCSI motifs of *Rh3* and *Rh5* should be interchangeable, just like their proximal core promoters. Surprisingly, the specific swap of the *Rh3* RCSI with the *Rh5* RCSI in the *Rh3* promoter context caused derepression in yR7s (Fig 7E and 7E’), which was not observed in the *Rh3*-*Rh5* hybrid. Therefore, unknown motifs or features in the proximal *Rh5* promoter region downstream of the RCSI prevented yR7/Rh4 subset expression in the hybrid.

In summary, the RCSI swap results provide further evidence for our model that the RCSI sequence is critical for generating the PR subtype-restricted pattern [19]. However, the different spatial outcomes after the swap of a particular RCSI motif or the entire proximal region (this study) demonstrate that the RCSI motif is not the only relevant proximal *cis*-regulatory element. Other proximal core motifs or features that are yet to be discovered (such as spacing to the TATA, GC content, phasing, etc.), play an additional role in generating the *Rh* patterns.

### Specific combinations of distal and proximal motifs generate the photoreceptor subtype-restricted patterns

**Fig 9:**
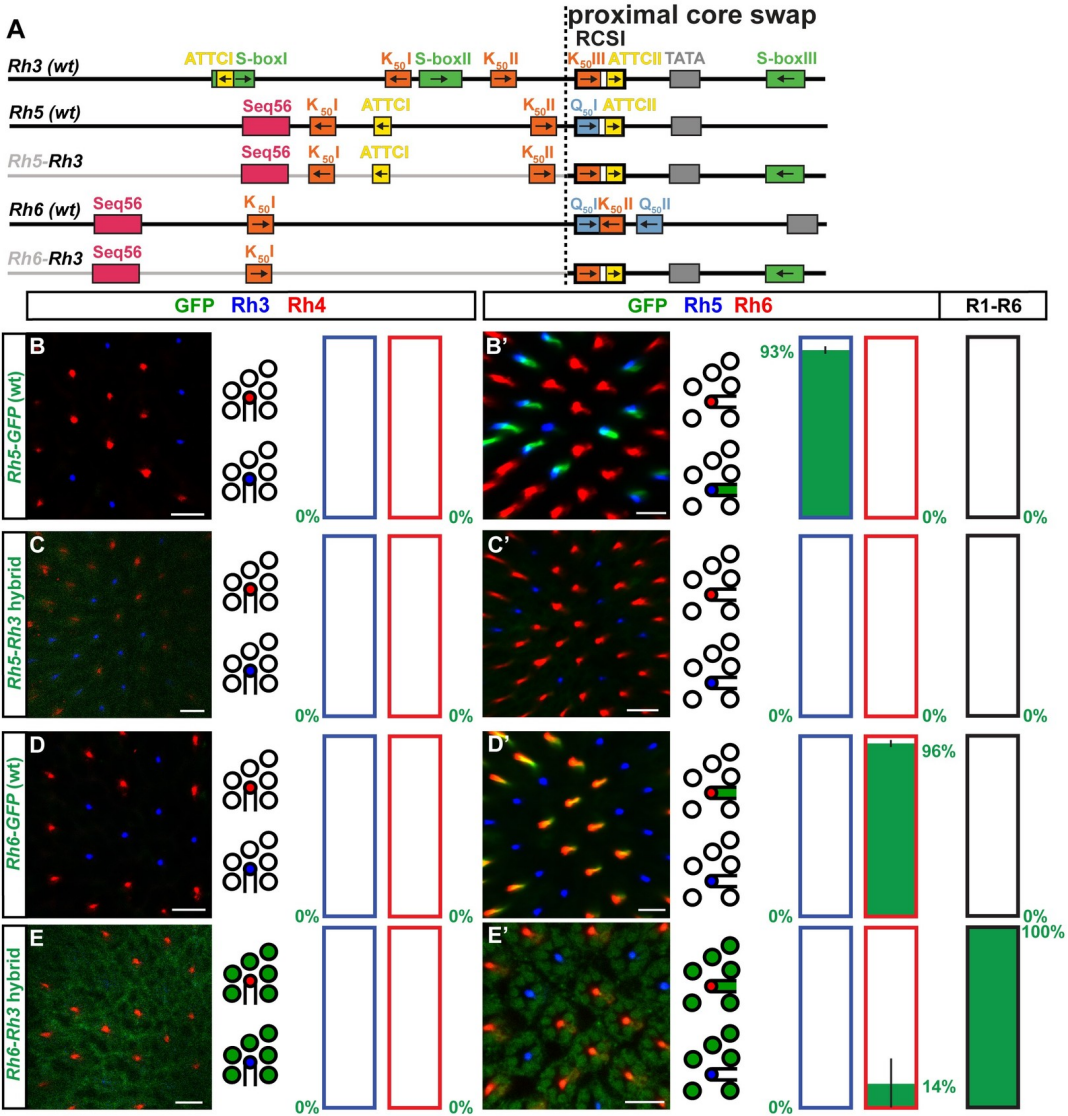
Shared *cis*-regulatory features do not predict the compatibility of distal and proximal promoter regions. (A) Schematic comparison of wild type promoters and hybrids to test the reverse compatibility of hybrids that had compatible motif combinations (see text). Note shared motifs such as K_50_ motifs and ATTC motifs. The dotted vertical lines indicate the break/fusion point of the hybrids immediately upstream of the RCSI motif that is found in a similar position in all *Rhs*. (B)—(E) Wild type promoters and hybrids driving GFP reporter expression (green) in the R7 layer. Rh3 (blue) labels pR7s and Rh4 (red) labels yR7s. Bar graphs show GFP co-expression in the Rh3 or Rh4 subset, respectively. (B’)—(E’) Wild type promoters and hybrids driving GFP reporter expression (green) in the R8 layer. Rh5 (blue) labels pR8s and Rh6 (red) labels yR8s. Bar graphs show GFP co-expression (green) in the Rh5, Rh6, or R1-R6 subset. Green numbers indicate the mean percentage of co-expressing photoreceptors, error bar represents standard error of the mean. (B) and (B’) The wild type *Rh5* promoter drives subtype-specific GFP reporter expression in the pR8/Rh5 subset (statistics are the same as in Fig 5D–5D’). (C) and (C’) The *Rh5*-*Rh3* hybrid does not drive detectable GFP expression in photoreceptors. N = 12 retinas and n = 732 R7s for (C); N = 9 retinas, n = 576 R8s and 3,456 R1-R6 PRs for (C’). (D) and (D’) The wild type *Rh6* promoter drives subtype-specific GFP expression in the yR8/Rh6 subset. N = 12 retinas and n = 786 R7s for (D); N = 12 retinas, n = 1,313 R8s and 7,878 R1-R6 PRs for (D’). (E) and (E’) The *Rh6*-*Rh3* hybrid drives weak GFP expression in R1-R6 and some yR8s. N = 7 retinas and n = 565 R7s for (E); N = 10 retinas, n = 690 R8s and 4,140 R1-R6 PRs for (E’). Scale bars, 10 μm.

To gain more insights into why only some distal and proximal motif combinations were compatible with driving a PR subtype-restricted pattern, we asked whether the compatibility of one particular hybrid combination of *Rhs* with shared motifs (e.g. *Rh3*-*Rh5*) allowed us to predict their compatibility in the reverse combination (e.g. *Rh5*-*Rh3*, Fig 9A). The ***Rh3*-*Rh5*** hybrid drove a PR subtype-specific expression in the pR7/Rh3 subset (see above and Fig 6E and 6E’) and thus resembled a wild type *Rh3* promoter. Consistent with this compatibility, the promoters of *Rh3* and *Rh5* share several features such as repeated K_50_ motifs, repeated ATTC motifs–one located within the same location of the RCSI–and the same close spacing of their RCSI to the TATA box (Fig 9A). However, the reverse ***Rh5*-*Rh3*** hybrid did not drive detectable reporter expression (Fig 9C and 9C’, compare to Fig 9B and 9B’). Moreover, while the ***Rh3*-*Rh6*** hybrid drove inner PR class-restricted expression (see above and Fig 6C and 6C’), the reverse ***Rh6*-*Rh3*** hybrid drove variable expression in a fraction of yR8s, but also in R1-R6 PRs (Fig 9E and 9E’, compare to wild type in Fig 9D and 9D’). Interestingly, the ***Rh5*-*Rh6*** hybrid revealed a partial compatibility of the distal and proximal promoter region because it drove incomplete expression in about half of the pR8/Rh5 PRs (S5B and S5B’ Fig). In contrast, the reverse ***Rh6-Rh5*** hybrid failed to drive detectable reporter expression (S5C and S5C’ Fig).

**Fig 10:**
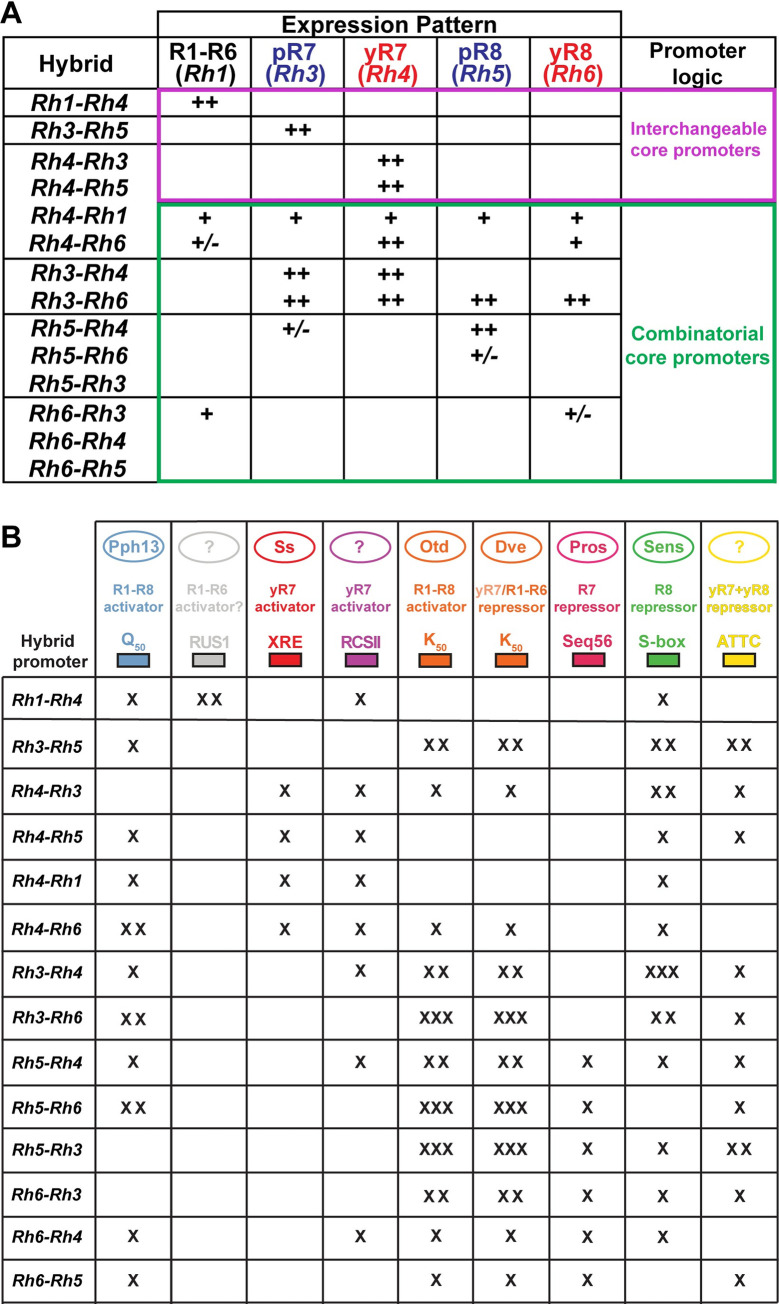
Summary of hybrid promoter driven expression patterns. (A) Summary of the spatial expression patterns of the hybrid promoters in five photoreceptor subtypes of the adult eye. Hybrids that followed the ‘interchangeable core’ model or ‘combinatorial core’ model (right column) are highlighted; note that few hybrid combinations gave perfectly restricted expression patterns in a single photoreceptor subtype (magenta), while most hybrids drove partial restriction in few photoreceptor subtypes or broad expression patterns (green). ‘++’ indicates strong expression, ‘+’ weak expression, ‘+/-’ variable expression. For details, see text. (B) Summary of transcriptional activators and repressors as well as the presence or absence of their corresponding *cis*-regulatory motifs in the hybrid promoters that were tested in this study. ‘x’ indicates that one motif was present in the hybrid promoter, ‘xx’ two motifs, ‘xxx’ three motifs.

Taking the results of the reverse hybrid tests together, two were non-functional (*Rh6-Rh5* and *Rh5-Rh3*), one partially labeled the subtype (*Rh5-Rh6*), and one generated a novel pattern (*Rh6-Rh3*). These outcomes are also consistent with the ‘combinatorial core’ model that is therefore supported by most of the hybrids that we tested in this study (Fig 10).

## Discussion

### Hybrid promoters as a tool to gain insights into the *cis*-regulatory logic of *rhodopsins*

Color vision requires the expression of Rhs with different wavelength sensitivities in different PR subtypes [1]. Previous studies [16,18–20] have identified sequence-specific transcription factors and *cis*-regulatory motifs in the *Rh* promoters that are required for the spatially precise *Rh* expression patterns. In this study, we investigated the roles of the distal and the proximal core promoter regions in directing the precise, PR subtype-restricted *Rh* expression patterns. As a complementary strategy to classic promoter mutagenesis that demonstrated the *requirement* of individual *cis*-regulatory motifs [17,18], we generated hybrid promoters to test the functional specialization of specific promoter regions and the *sufficiency* of novel motif combinations for generating a *Rh*-like pattern.

In contrast to synthetic promoters that are used to test combinations of isolated motifs that are spaced by random sequences, the hybrids test the motifs in their original (distal or proximal core promoter) environment and thus include other *cis*-regulatory information (e.g. spacing, GC content, phasing, etc.) as well. This is advantageous because the synthetic reconstruction approach has been unsuccessful even for the best-understood developmental enhancers [21], most likely because we do not know all the *cis*-regulatory rules (sometimes called ‘grammar’) that define a functional *cis*-regulatory region. In this respect, replacement of larger regions in hybrids that reveal compatibilities or incompatibilities of motif combinations can provide valuable information to narrow down the minimal elements of a functional promoter. An example from the current study are the different spatial outcomes of RCSI swaps compared to the replacements of the corresponding proximal core region.

### Hybrid promoters give insights into the combinatorial motif architecture that controls *rhodopsin* expression

We generated hybrid promoters and swapped RCSI motifs to distinguish between two models of the roles of the proximal core promoter. Only four hybrids (*Rh1-Rh4*, *Rh3-Rh5*, *Rh4-Rh3*, and *Rh4-Rh5*) generated subtype-specific expression patterns that resembled the expression patterns of the *Rh* that provided the distal promoter region (Fig 10A). These hybrids were consistent with the original ‘bipartite promoter’ and ‘interchangeable core’ models that predict that only the distal region is critical for generating the PR subtype-specific pattern and that the proximal region is interchangeable. However, most hybrids (10/14) did not fit these two models but yielded different outcomes (Fig 10A) (for detailed interpretations and discussion of the transcription factors involved, see S1 Text). One hybrid (*Rh5-Rh6*) was partially functional and drove incomplete reporter expression in a fraction of a subtype, while three (*Rh5*-*Rh3*, *Rh6-Rh4*, and *Rh6-Rh5*) failed to drive any expression in photoreceptors. These activation defects can either be interpreted as an incompatibility of the distal and proximal motifs to provide combinatorial activation or as a result of repression that prevented activation. One hybrid (*Rh3-Rh4*) drove a novel expression pattern that resembled the combination of the expression patterns of the *Rhs* that contributed the distal and proximal region, suggesting that both regions contributed to the expression pattern. In contrast, five hybrids drove broadened expression patterns in two or more PR subtypes that were not a simple combination of the contributing *Rhs*’ expression patterns (*Rh3-Rh6*, *Rh4-Rh1*, *Rh4-Rh6*, *Rh5-Rh4*, and *Rh6-Rh3*). Together with the RCSI swap results, which are consistent with our previous model [19] that the specific sequence of the RCSI motif is critical for subtype-specific *Rh* expression, all these data suggest that the distal and the proximal core promoter motifs need to be precisely matched to generate perfectly subtype-specific patterns. Although only few hybrids could be reconciled with the original ‘bipartite promoter’ model, it was nevertheless a useful working model that guided the discovery of key *cis*-regulatory motifs and *trans*-acting factors such as K_50_/Otd/Dve and Seq56/Pros [8,13] (Fig 10B). Furthermore, we propose below that the ‘bipartite promoter’ model might represent an ancestral *Rh* promoter signature.

### The evolution of the *cis*-regulatory signatures of the *Drosophila rhodopsins*

**Fig 11:**
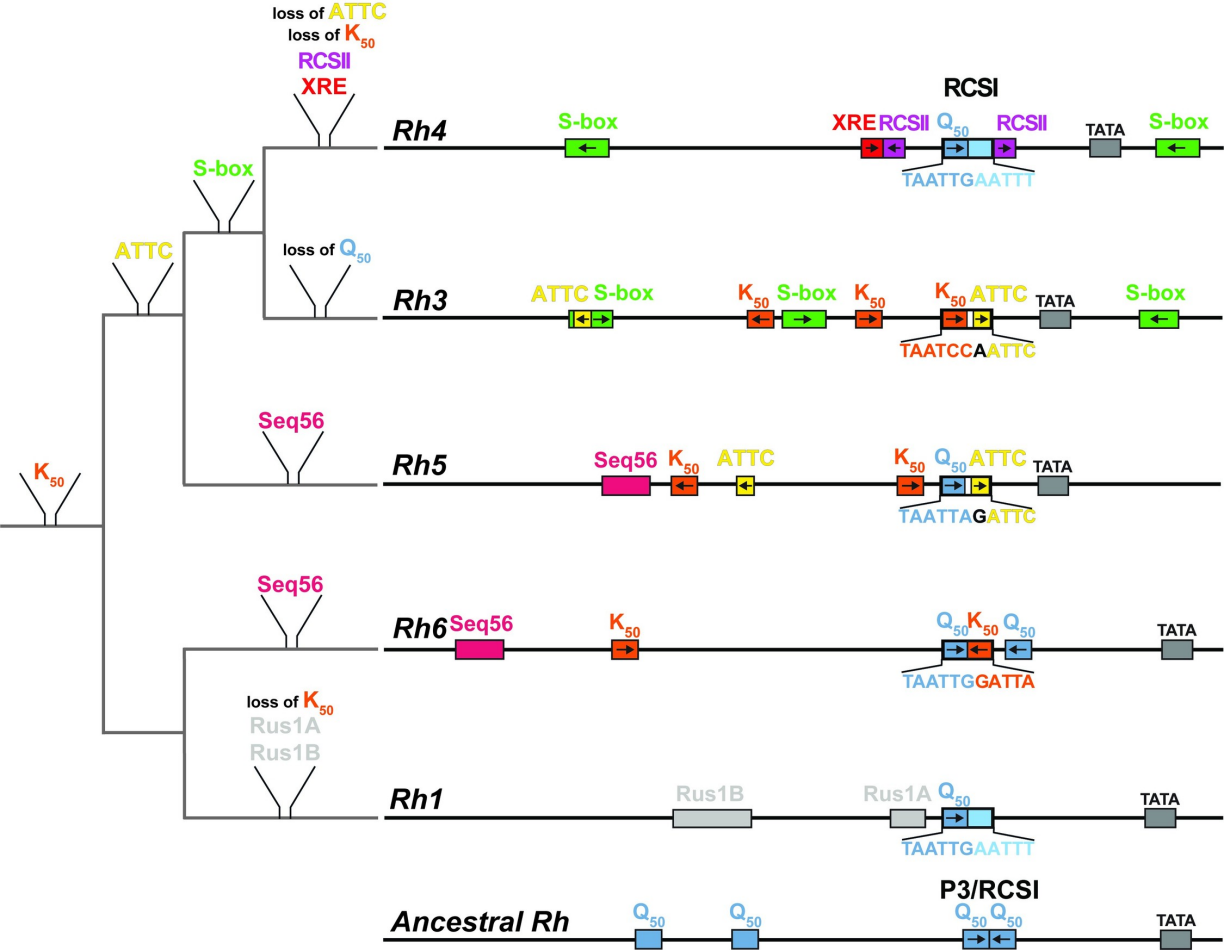
Evolution of the *rhodopsin* promoter *cis*-regulatory signatures. Model of the *cis*-regulatory evolution during the subfunctionalization of duplicated *rhodopsin* genes. Branch length does not reflect degree of sequence divergence. Left: phylogenetic tree (grey) with proposed motif gains or losses based on the evolutionary relationships of the coding sequences of the different *rhodopsin* genes as well as motif similarities (colored) in the contemporary promoters (right). Right: promoters of the five contemporary *rhodopsins* that are expressed in the adult eye. Note motif similarities among the *Rh6*, *Rh5*, and *Rh3* promoters as well as the substantial divergence of the *Rh1* and *Rh4* promoters. Bottom: the proposed ancestral *Rhodopsin* promoter has distal and proximal Q_50_ motifs for broad activation. Its proximal promoter region contains a palindromic P3-type motif, while each contemporary *rhodopsin* promoter has a specific RCSI motif (top).

The replacement of larger promoter regions, which we performed to obtain the hybrid combinations, is unlikely to be the mechanism for how the *cis*-regulatory logic of *Rh* expression evolved. However, the hybrid results gave us valuable insights into how optimal motif combinations might have evolved through the addition or loss of motifs in the distal or proximal core promoter region. The *Drosophila Rh* genes arose from duplications of a single ancestral *Rh* gene [22,23] and their subsequent subfunctionalization [24] through specific coding sequence mutations that resulted in different wavelength sensitivities [25,26]. We propose that the *Rh* gene duplications also included the promoter region, whose mutation generated novel *cis*-regulatory motifs for specific *trans*-acting factors that partition the spatially distinct *Rh* patterns. Taking both the phylogenetic relationships of the *Rh* coding sequences [22,23] as well as shared *cis*-regulatory motifs in their promoters into account, we suggest the following model for the evolution of the *Rh cis*-regulatory signatures (Fig 11): In agreement with previous models [19,22,27], the ancestral *Rh* was probably broadly expressed by the ancient pan-PR activator Pph13 [28] through Q_50_ motifs in the distal promoter region and within a palindromic P3-type RCSI motif in the proximal region (Fig 11). Such a palindromic P3-type motif, composed of Q_50_ motifs and lacking repressor motifs (Fig 11), closely resembles the palindromic P3 motifs in the contemporary Pph13-dependent phototransduction genes that are also broadly expressed in all PRs [6,19].

The duplication of the single ancestral *Rh*, which encoded a long wavelength-sensitive Rh, generated two major lineages: the first lineage includes the long wavelength-sensitive Rh6/Rh1/Rh2 (*Rh2’s* regulation is not discussed here because it is less well understood) and the second lineage includes the short-wavelength sensitive Rh5/Rh4/Rh3 [22,23,29] (Fig 11). It is possible that the *cis*-regulatory divergence of the first duplicated *Rh* genes was largely driven by mutations in the distal promoter region and that the proximal core’s function remained to provide ‘generic’ activation in all PRs by a palindromic P3-type motif. This view is consistent with the larger number of *cis*-regulatory motifs in the distal region and would conceptually be in agreement with the ‘bipartite promoter’ and ‘interchangeable core’ models; these two models would thus represent an ancestral *cis*-regulatory signature.

Following the duplication of their precursor gene, the spatial distinction of the expression patterns of *Rh6* (in yR8s) and *Rh1* (in R1-R6) required a substantial modification of the ancestral ‘generic activation’ signature. In the case of *Rh6*, this involved a switch from Q_50_ to K_50_ motifs that differ in only two base pairs [9,12,19] for Dve-mediated repression in R1-R6 as well as the gain of a Seq56 motif for Pros-mediated repression in R7s [13] (Fig 11). The corresponding mechanisms in *Rh1* have yet to be identified.

Another duplication event separated *Rh5* from *Rh3*/*Rh4* [25,30] and a subsequent retrotransposition event generated the intronless *Rh3* from the *Rh3*/*Rh4* ancestor [31]. The distal motif similarities of *Rh5*, *Rh6*, and *Rh3* match their phylogenetic coding sequence relationships (Fig 11): *Rh5* and *Rh6* share a Seq56/Pros motif, K_50_/Otd/Dve motifs, and a Q_50_/Pph13 motif that mediate their repression in R1-R7s and broad activation. While *Rh6* and the more recently evolved *Rh3* only share K_50_ motifs, *Rh5* and *Rh3* share K_50_ motifs as well as ATTC/y repressor motifs. In contrast, a substantial *cis*-regulatory divergence occurred between the closely related *Rh4* and *Rh3* (Fig 11) that are expressed in subsets of R7 PRs, but their promoters only share a distally and a proximally located S-box motif for R8 repression. We suggest that *Rh4* lost the K_50_ motifs–which would be incompatible with yR7 expression due to the yR7 repressor Dve–and the K_50_/Otd-dependent activation [8] was replaced by a unique XRE activator motif that is bound by the yR7-specific activator Ss [10,11].

The increased regulatory complexity after several *Rh* gene duplications, the partitioning of five *Rhs* in the retinal mosaic, and the division into two subtypes of R7s (pR7/Rh3 and yR7/Rh4) and R8s (pR8/Rh5 and yR8/Rh6) required a modification of the ancestral proximal promoter signature. Consistent with this rationale, *Rh6*, *Rh5*, and *Rh3* exhibit specific modifications in their RCSI motifs including repressor motifs that are required for full subtype specificity ([19] and this study). It is likely that the evolution of *cis*-regulatory mechanisms that ensure the precise and perfectly subtype-specific *Rh* expression patterns occurred in a stepwise manner through imperfect patterns with partial restriction, similar to the motif combinations in the hybrid series *Rh3*-*Rh6*, *Rh3*-*Rh4*, and *Rh3*-*Rh5* (see above).

### Comparison of the *cis*-regulatory signatures of *rhodopsins* with olfactory genes and other regulatory contexts

Like PR neurons that express a specific *Rh* gene, *Drosophila* olfactory receptor (OR) neurons of the antenna or the maxillary palp express a specific OR gene [4,32]. Yet, with its larger repertoire of 60 OR genes [33], the *Drosophila* olfactory system represents a much greater regulatory challenge than the eye that expresses five *Rhs*. This difference in complexity is reflected in the *cis*-regulatory signatures. The OR gene signatures do not show a *Rh* promoter-like distal-proximal motif separation with a shared RCSI-like motif, but are based on structured motif clusters [34]. Whether the transcription factors that bind these motifs exhibit activating or repressing activity depends on the motif location with respect to the TATA box [34,35]. The motif arrangement is therefore critical for the OR expression pattern, similar to what has been described for embryonic enhancers [36].

The olfactory receptor gene promoters contain clusters of low affinity homeodomain motifs, which might promote transcription factor cooperativity or competition at overlapping motifs to achieve restricted expression patterns [34]. This is reminiscent of the predominant use of low affinity homeodomain motifs to recruit specific Hox protein transcription factors in the *Drosophila* embryo [37] or to shape a morphogen response in specific tissues [38]. In contrast to this preference for low affinity motifs, *Rhs* contain multiple high-affinity homeodomain motifs (K_50_ and Q_50_) in their distal and proximal promoter regions. These highly conserved motifs are bound by two ancestral transcription factors (Otd and Pph13, respectively) that are broadly expressed in all photoreceptors and also activate broadly expressed phototransduction genes [6,19,39]. Very similar Q_50_ motifs and K_50_ motifs–the latter bound by the Otd ortholog Crx [40]–are enriched in mammalian rod and cone PR genes [41]. The mammalian PR genes also contain dimeric Q_50_ motifs that resemble the RCSI motif (consensus: TAATYNRATTN) and the related P3 motif (consensus: TAATYNRATTA), which is found in the promoters of broadly expressed phototransduction genes in *Drosophila* [42]. A similar role of multiple copies of terminal selector motifs [43] in providing robustness has been described in *C*. *elegans* motor neuron differentiation [44]. The use of evolutionarily conserved redundant motifs that increase the robustness of gene expression has also been described in embryonic enhancers [45].

High-affinity homeodomain motifs allow *Rhs* to achieve competition by ‘taking advantage’ of the mere two base pair difference between Q_50_ (TAATTG/A) and K_50_ (TAATCC) motifs. This distinction required minor evolutionary changes and permitted the recruitment of different *trans*-acting factors with opposing activities (Pph13 activator *vs*. Otd activator/Dve repressor) [9,12,19]. A reason for the predominance of high-affinity motifs in *Rh* promoters could thus be that only high-affinity motifs reliably recruit the repressor Dve, which then outcompetes the activator Otd, as has been demonstrated in cell culture [12].

The larger number of options to generate low affinity motifs and the related flexibility appear to make the expression of essential embryonic genes more robust [46]. *Rhs* might not need this buffer because they are not absolutely required for survival. An advantage of high-affinity motifs is that they seem to be more suitable for establishing and maintaining the extremely high *Rh* expression levels (www.flyatlas.org) [47]. However, basing the precise *Rh* patterns on high-affinity motifs leads to constraints in the combinatorial logic and appears to be less flexible than the generation of expression patterns with low affinity motifs.

Transcriptional repression plays a key role for generating the PR subtype-specific *Rh* expression patterns. Likewise, repression is critical for restricting an OR gene to the correct olfactory sensory neuron type [35,48,49] and the distinction of sensory and motor neuron subtypes in *C*. *elegans* [50–52]. It is therefore an important experimental goal to decipher the underlying mechanisms that distinguish broad from restricted, cell-type specific gene expression patterns. The complexity of this task is illustrated by the finding that it is not trivial to computationally predict, based on identified promoter features, whether a resulting expression pattern will be broad or tissue-specific [53].

Like in *Rh* promoters, motif sharing is common among the OR gene promoters: a motif has been identified (Oligo-1 motif) that plays a dual role in both activation and repression [35,48,49], like the K_50_ motifs in the *Rh* promoters. However, there is also some evidence for the use of OR gene-specific, i.e. unique regulatory motifs [49]–comparable to the XRE/Ss motif in *Rh4* –for OR genes that are expressed in OR neurons of the maxillary palp.

In conclusion, to further our understanding of how sensory neuron diversity is generated and how it evolved, the analysis of a larger number of promoters and enhancers in various sensory contexts would be highly desirable [54]. A deep understanding of the underlying mechanisms could eventually inform targeted medical applications to generate specific neuron types or to develop gene therapies for disorders that affect specific neuron types.

## Materials and methods

### Cloning of *rhodopsin* promoter hybrids and RCSI swap constructs

To generate hybrid *Rh* promoters, we PCR-amplified the proximal region of one *Rh* promoter and the distal region of another *Rh* promoter with the Expand High Fidelity PCR System (Roche) from pGEM T-Easy plasmids (Promega) that contained the respective minimal *Rh* promoters (see primer sequence Table 1). We stitched the amplified distal and proximal promoter regions together by PCR-driven overlap extension/’PCR sewing’ [55]. Full sequences of the hybrid promoters are available upon request. To generate RCSI swaps, we introduced point mutations in the minimal *Rh* promoters (see Tables 1 and 2 with primer sequences below) using the QuikChange site-directed mutagenesis kit (Stratagene). Lastly, we confirmed all hybrids and RCSI swaps by Sanger sequencing.

**Table 1:** Primers for generating minimal *rhodopsin* promoters and hybrid promoters. The minimal *rhodopsin* promoters have been previously described [19]; the underlined sequences are *BglII* or *NotI* restriction sites that we used for cloning.

*Rhodopsin*	Forward primer	Reverse primer
*Rh1*	agatctataatccaagattagcagag	gcggccgcattctgaatatttcactggg
*Rh3*	agatctgcactaaccttcagatgagc	gcggccgcgtctgcgggccaagacgaaatca
*Rh4*	agatctgtttctttggacaatgttg	gcggccgctcggtcaacccgataccgaa
*Rh5*	agatctaacatgtaaagcttgtaaaa	gcggccgctagtttcctttgcaggtcgac
*Rh6*	agatctgggtgggtggtacctcaaac	gcggccgcggtggcgcttcggtggtggcttc

**Table 2:** Primers for generating RCSI and RCSII swaps. We performed the RCSI and RCSII swaps on the minimal *rhodopsin* promoters [19].

RCSI swap	Forward primer	Reverse primer
*Rh3-*RCSIIswap*-Rh4*	acaatgctaatccaattcggttgcgatgggccgtataaaa	ttttatacggcccatcgcaaccgaattggattagcattgt
*Rh3*-RCSI+IIswap-*Rh4*	tcccgctgcgacaatgctaattgaatttggttgcgatgggccgtataaaa	ttttatacggcccatcgcaaccaaattcaattagcattgtcgcagcggga
*Rh3*-RCSIswap*-Rh5*	taatcccgctgcgacaatgctaattagattccgatgggccgtataaaagc	gcttttatacggcccatcggaatctaattagcattgtcgcagcgggatta
*Rh3*-RCSIswap*-Rh6*	taatcccgctgcgacaatgctaattggattacgatgggccgtataaaagc	gcttttatacggcccatcgtaatccaattagcattgtcgcagcgggatta
*Rh4*-RCSIswap*-Rh3*	aaccacaaagtctaatccaattcggttggcagcacaaaatgcgat	atcgcattttgtgctgccaaccgaattggattagactttgtggtt
*Rh4*-RCSIswap*-Rh6*	aaccacaaagtctaattggattaggttggcagcacaaaatgcgat	atcgcattttgtgctgccaacctaatccaattagactttgtggtt
*Rh5*-RCSIswap*-Rh3*	ggtcaccacttaatccgtcttaatccaattctttggcgggctataaaagc	gcttttatagcccgccaaagaattggattaagacggattaagtggtgacc
*Rh5*-RCSIswap*-Rh6*	tcaccacttaatccgtcttaattggattatttggcgggctataaaagca	tgcttttatagcccgccaaataatccaattaagacggattaagtggtga
*Rh6*-RCSIswap*-Rh3*	ggccaagtgccggctaatccaattcgggcaattagtcta	tagactaattgcccgaattggattagccggcacttggcc
*Rh6*-RCSIswap*-Rh5*	ggccaagtgccggctaattagattcgggcaattagtcta	tagactaattgcccgaatctaattagccggcacttggcc

### Generation and maintenance of transgenic animals

We inserted the hybrid and RCSI swap promoters into a transformation plasmid containing an *egfp* reporter gene, a *mini-white*^*+*^ transformation marker and an *attB* site for *phiC31*-mediated transgenesis [19]. Next, we injected the transformation plasmid into *white*-mutant *Drosophila* embryos that expressed phiC31 integrase in the germ line and carried the third chromosomal landing site J36 (*ZH-attP-86Fb*) [56]. We crossed the resulting adult flies to *white*-eyed balancer flies (*yw*^*67*^, *hsflp*; *Sp*/*CyO*; *TM2*/*TM6*) and screened the offspring for *white*^*+*^ as a marker for successful integration of the transgene. Next, we established stably balanced stocks and maintained them on standard medium at 25°C under a 12 hour/12 hour light/dark cycle.

### Immunohistochemistry and confocal microscopy

We performed immunohistochemistry as previously described [57]. Briefly, we dissected adult retinas of female flies that were homozygous for the reporter construct in cold phosphate-buffered saline (PBS) and fixed in 3.7% formaldehyde solution for 15 minutes at room temperature, followed by two washes with PBS and one with PBST (PBS + 0.2% Triton-X, Sigma). Next, we removed the laminas and incubated the retinas overnight with the following primary antibodies that were diluted in PBST: sheep anti-GFP (1:100, AbD Serotec), mouse anti-Rh3 (1:10) or mouse anti-Rh5 (1:400, both antibodies were a gift from S. Britt, University of Texas at Austin), and guinea pig anti-Rh4 (1:1000) or rabbit anti-Rh6 (1:1000, both antibodies were a gift from C. Desplan, New York University). The next morning, we performed three PBST washes and then incubated the retinas in the secondary antibodies (Alexa Fluor 488-, 555-, or 647-conjugated raised in donkey; Molecular Probes), which were diluted 1:800 in PBST, overnight at room temperature. Three washes in PBST followed. We mounted the retinas on bridge slides with SlowFade (Molecular Probes) and imaged them with Leica SP5 and Zeiss LSM 8 confocal microscopes. We processed the confocal images with Leica LAS AF Lite, Fiji [58], Adobe Photoshop 2020, and Adobe Illustrator 2020 software. The same contrast settings were used for wild type and mutant reporter constructs.

### Quantification of reporter expression patterns

The number of rhabdomeres that expressed Rhodopsin antibody markers and the GFP reporter was manually scored (using the count tool in Adobe Photoshop 2020), based on the presence or absence of detectable antibody signal, in two to four days old female flies. For quantification of R7 rhabdomeres, only the central and ventral retina were scored to prevent confusion of the regional co-expression of Rh3 and Rh4 in dorsal third yR7 ommatidia [59] with GFP reporter derepression in yR7s. 7 to 18 retinas (N) were scored per reporter construct; the specific number of rhabdomeres (n) that were scored are provided in the figure legends. Bar graphs represent the number of rhabdomeres that were positive for the respective Rh antibody (the endogenous PR subtype marker) normalized to 100%, as well as the average number of rhabdomeres expressing the GFP reporter in the same PR subtype. Error bars depict the standard error of the mean (s.e.m.). Statistical comparisons to wild type promoters were performed using the Mann-Whitney U Test and significance levels are represented as p values.

### Conservation analysis of *cis*-regulatory motifs

To analyze the evolutionary conservation of *cis*-regulatory motifs and their variants, we obtained alignments of the *rhodopsin* promoter regions of 12 sequenced *Drosophila* species [60] from the UCSC genome browser (https://genome.ucsc.edu/). Dashes in the alignments represent gaps and double dashes represent a lack of corresponding (‘alignable’) sequences. We identified orthologous genomic sequences using BLAT (https://genome.ucsc.edu/cgi-bin/hgBlat) [61]. The output was copied to Microsoft Word; we analyzed the alignments for inconsistencies and manually adjusted them to correct local misalignments of conserved motifs. Schematics representing motif conservation were redrawn using Adobe Illustrator 2020.

### Motif variant scoring

We counted the frequencies of occurrence of K_50_ and Q_50_ motif variants in the *rhodopsin* promoters of the 12 *Drosophila* species. To determine the affinity of Otd and Pph13 for each motif variant, respectively, we obtained position-weight matrices (PWM) for Otd and Pph13 binding sites from FlyFactorSurvey (https://mccb.umassmed.edu/ffs/) [62]. PWM similarity scores were generated for the entire motif sequence by summing up the weighted values for each mononucleotide. To normalize for the different number of base pairs of different motifs, we calculated percentages relative to the highest-scoring base pair configuration for a given PWM, with the strongest motif represented as 100. Positive PWM scores thus indicate a high probability that a given sequence is a functional binding site, whereas negative scores indicate a non-functional or random site.

## Supporting information

S1 Fig Combinations and function of evolutionarily conserved *cis*-regulatory motifs. Schematics of the alignments of the *rhodopsin* promoter sequences of 12 *Drosophila* species. Motif lengths and spacing are to scale; arrows indicate motif orientation. White lines indicate differences between species and thus lack of evolutionary conservation. (A) The *Rh4* promoter drives specific expression in yR7s (right). It has a unique and perfectly conserved XRE motif and a less conserved *Rh4* RCSII motif for activation in yR7s, as well as two S-box motifs for repression in R8. The highly conserved *Rh4* RCSI contains a Q_50_ homeodomain motif that is inverted in distant species and provides broad activation in all photoreceptors. (B) The *Rh3* promoter drives specific expression in pR7s (right). It has three highly conserved K_50_ motifs (one within the *Rh3* RCSI) that are shared with *Rh5* and *Rh6* and provide broad activation in all photoreceptors. The K_50_ motifs also mediate repression in R1-R6 and yR7s, the latter in support with the two highly conserved ATTC motifs (one within the *Rh3* RCSI). The three S-box motifs show variable conservation and mediate R8 repression. Note the overlap of ATTCI and S-boxI. (C) The *Rh5* promoter drives specific expression in pR8s (right). The conserved Seq56 motif provides R7 repression. *Rh5* also has two K_50_ motifs for broad activation and R1-R6/yR7 repression; the distal one slightly changes its position and orientation in distant species. The two ATTC motifs (one within the *Rh5* RCSI) mediate yR8 repression, while the Q_50_-motif in the *Rh5* RCSI mediates broad activation in all photoreceptors. (D) The *Rh6* promoter drives specific expression in yR8s (right). It shares the Seq56, the two K_50_ motifs (one within the *Rh6* RCSI), and the Q_50_ motif (within the *Rh6* RCSI) with *Rh5*. Note the second Q_50_ motif downstream of the *Rh6* RCSI as well as the perfect conservation of the K_50_ and the Q_50_ motifs.
(EPS)

S2 Fig Conservation analysis of *cis*-regulatory motifs in different *rhodopsin* promoters. (A) The *Rh4* promoter contains a unique and perfectly conserved distal XRE motif as well as a less well conserved *Rh4* RCSII motif for activation in yR7s. The two S-box motifs for repression in R8 are less well conserved in distant species. The highly conserved *Rh4* RCSI motif contains a high-affinity Q_50_ homeodomain motif–the three variants are shown to the right–that is inverted in distant species. Scores on the right are matrix similarity scores for each motif variant for the activator Pph13 that binds Q_50_ motifs (maximal score is 100) and number of motif occurrences in all 12 *Drosophila* species. (B) The *Rh3* promoter has three highly conserved K_50_ motifs (one within the *Rh3* RCSI) that occur in two high-affinity variants (shown to the right). The two proximal ones are almost perfectly conserved, while the distal motif shows more variation and is lost in two species. The three S-box motifs show a high level of conservation of their AATC cores. Scores on the right are matrix similarity scores for each motif variant for the activator Otd that binds K_50_ motifs (maximal score is 100) and number of motif occurrences in all 12 *Drosophila* species. (C) Like *Rh3*, the *Rh5* promoter has highly conserved K_50_ motifs and the distal one shows more variability, including a slight position shift and sequence inversion. There are three motif variants, but the highest affinity motif is much more common; the two weaker motif variants occur only once and thrice, respectively. The distal ATTCI motif is also much less conserved than the perfectly conserved proximal ATTCII that is part of the *Rh5* RCSI. The Seq56 motif is less conserved in distant species. The Q_50_-motif in the *Rh5* RCSI is perfectly conserved and is the highest affinity variant. Scores on the right are matrix similarity scores for each motif variant for the activators Otd and Pph13 that bind K_50_ motifs and Q_50_ motifs (maximal score is 100), respectively. Also indicated is the number of motif occurrences in all 12 *Drosophila* species. (D) The *Rh6* promoter’s Seq56 is similarly conserved as the one in *Rh5*. The two K_50_ motifs and the two Q_50_ motifs, one within the *Rh6* RCSI and the other one downstream, are perfectly conserved and are all high-affinity motifs. Scores on the right are matrix similarity scores for each motif variant for the activators Otd and Pph13 that bind K_50_ motifs and Q_50_ motifs (maximal score is 100), respectively. Also indicated is the number of motif occurrences in all 12 *Drosophila* species. (E) Top: The *Rh1* promoter has highly conserved distal Rus1B and Rus1A motifs, as well as a highly conserved RCSI that is very similar to *Rh4*’s (see A) but is never inverted. The Q_50_ motif in the *Rh1* RCSI is a high-affinity motif in 11/12 species. Scores on the right are matrix similarity scores for the activator Pph13 that binds Q_50_ motifs (maximal score is 100) and the number of motif occurrences in all 12 *Drosophila* species. Bottom: schematic of motif conservation that also highlights the conserved motif positions and orientations. Base pair (‘bp’) indications below vertical lines of the motif alignments indicate the number of base pairs that were omitted to better display key *cis*-regulatory motifs. Sequences shown in the alignments are from the following *Drosophila* species (from top to bottom): *D*. *melanogaster*, *D*. *simulans*, *D*. *sechellia*, *D*. *yakuba*, *D*. *erecta*, *D*. *ananassae*, *D*. *pseudoobscura*, *D*. *persimilis*, *D*. *willistoni*, *D*. *virilis*, *D*. *mojavensis*, and *D*. *grimshawi*.
(EPS)

S3 Fig The *Rh4* RCSI swap with the *Rh3* RCSI causes derepression. (A) Wild type (*wt*) *Rh4* promoter and specific swap of its RCSI with the RCSI of *Rh3* (indicated by dotted vertical lines). (B) The swap of the *Rh4* RCSI with the *Rh3* RCSI in the *Rh4* promoter context causes weak GFP derepression in a substantial fraction of the pR7/Rh3 subset (arrows). Rh3 (blue) labels pR7s and Rh4 (red) labels yR7s. Bar graphs show GFP co-expression (green) in the Rh3 or Rh4 subset, respectively. Green number indicates the mean percentage of co-expressing photoreceptors, error bar represents standard error of the mean. N = 9 retinas and n = 537 R7s. (B’) The swap of the *Rh4* RCSI with the *Rh3* RCSI in the *Rh4* promoter context does not cause reporter expression in the pR8/Rh5 (blue) yR8/Rh6 (red), or R1-R6 subset. Bar graphs show GFP co-expression (green) in the Rh5, Rh6, or R1-R6 subset. Green numbers indicate the mean percentage of co-expressing photoreceptors, error bar represents standard error of the mean. N = 9 retinas, n = 650 R8s and 3,900 R1-R6 PRs. Scale bars, 10 μm.
(TIF)

S4 Fig Incompatibility of distal *Rh6* and proximal *Rh4*. (A) Schematic comparison of the wild type *Rh4* promoter and the *Rh6-Rh4* hybrid. The dotted vertical line indicates the break/fusion point of the hybrid immediately upstream of the RCSI motif that is found in a similar position in all *Rhs*. (B)—(C) and (B’)—(C’) Hybrid and RCSI swap driving GFP reporter expression (green) in the R7 layer. Rh3 (blue) labels pR7s and Rh4 (red) labels yR7s. (D)—(E) and (D’)—(E’) Hybrid and RCSI swap driving GFP reporter expression (green) in the R8 layer. Rh5 (blue) labels pR8s and Rh6 (red) labels yR8s. (B) and (C) The *Rh6-Rh4* hybrid does not drive detectable GFP expression in R7 photoreceptors. 4/11 retinas exhibit faint reporter expression in pigment cells. N = 11 retinas and n = 1,454 R7s. (D) and (E) The *Rh6-Rh4* hybrid does not drive detectable GFP expression in R8 or R1-R6 photoreceptors. 5/11 retinas exhibit reporter expression in pigment cells. N = 11 retinas, n = 830 R8s and 4,980 R1-R6 PRs. (A’) Schematic comparison of the wild type *Rh6* promoter and the specific RCSI swap with the *Rh4* RCSI (indicated by the dotted vertical lines). Note the shared Q_50_ motif in the RCSI. (B’) and (C’) The swap of the *Rh6* RCSI with the *Rh4* RCSI in the *Rh6* promoter context does not drive detectable GFP expression in R7 photoreceptors. 2/10 retinas exhibit faint reporter expression in pigment cells. N = 10 retinas and n = 1,089 R7s. (D’) and (E’) The swap of the *Rh6* RCSI with the *Rh4* RCSI in the *Rh6* promoter context does not drive detectable GFP expression in R8 or R1-R6 photoreceptors. 1/10 retinas exhibit faint reporter expression in pigment cells. N = 10 retinas, n = 654 R8s and 3,924 R1-R6 PRs. Scale bars, 10 μm.
(TIF)

S5 Fig Shared *cis*-regulatory features do not predict the compatibility of distal and proximal promoter regions. (A) Schematic of wild type (*wt*) *Rh5* and *Rh6* promoters and hybrids to test the reverse compatibility of hybrids that had compatible motif combinations (see text). Note the shared K_50_ motifs and the Seq56 motif. The dotted vertical lines indicate the break- and fusion-points of the hybrids and RCSI swaps. (B) and (C) Hybrid promoter driven GFP reporter expression (green) in the R7 layer. Rh3 (blue) labels pR7s and Rh4 (red) labels yR7s. Bar graphs show GFP co-expression in the Rh3 or Rh4 subset, respectively. Green numbers indicate the mean percentage of co-expressing photoreceptors, error bar represents standard error of the mean. (B’) and (C’) Hybrid promoter driven GFP reporter expression (green) in the R8 layer. Rh5 (blue) labels pR8s and Rh6 (red) labels yR8s. Bar graphs show co-expression of GFP (green) in the Rh5, Rh6, or R1-R6 subset. Green numbers indicate the mean percentage of co-expressing photoreceptors, error bar represents standard error of the mean. (B) and (B’) The *Rh5*-*Rh6* hybrid drives incomplete GFP expression in a fraction of the pR8 subtype. N = 12 retinas and n = 807 R7s for (B); N = 10 retinas, n = 1,364 R8s and 8,184 R1-R6 PRs. (C) and (C’) The *Rh6*-*Rh5* hybrid does not drive detectable GFP reporter expression. N = 12 retinas and n = 858 R7s for (C); N = 8 retinas, n = 773 R8s and 4,638 R1-R6 PRs for (C’). Scale bars, 10 μm.
(TIF)

S1 Text Analysis of the usage of high-affinity homeodomain motifs and interpretations of the hybrid promoter expression patterns. (DOCX)
