# Supplementary figures and images for: A combinatorial cis-regulatory logic restricts color-sensing Rhodopsins to specific photoreceptor subsets in Drosophila
Source: PLoS Genet. 2021 Jun 23;17(6):e1009613. doi: 10.1371/journal.pgen.1009613 (PMC8259978; doi:10.1371/journal.pgen.1009613)

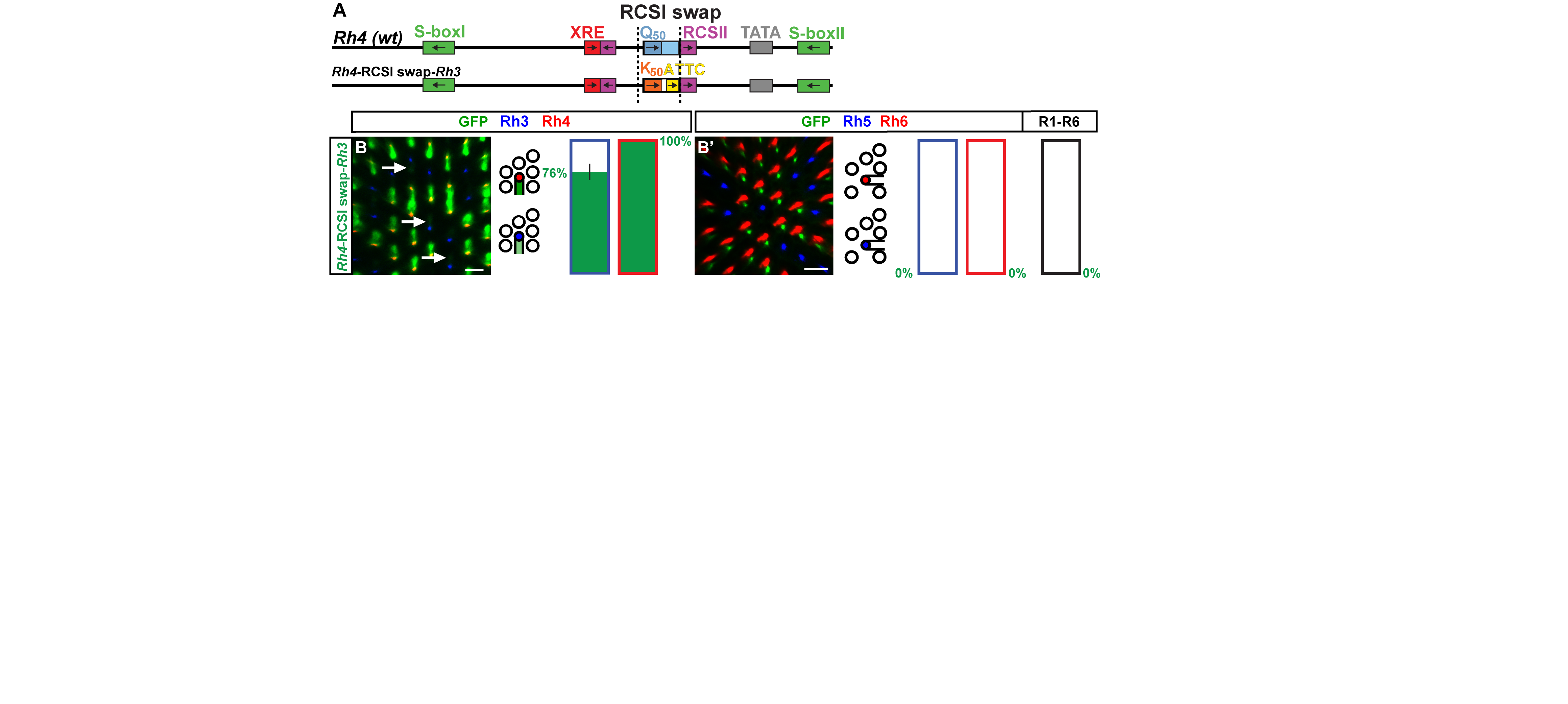

Supplement: S3 Fig — (A) Wild type (wt) Rh4 promoter and specific swap of its RCSI with the RCSI of Rh3 (indicated by dotted vertical lines). (B) The swap of the Rh4 RCSI with the Rh3 RCSI in the Rh4 promoter context causes weak GFP derepression in a substantial fraction of the pR7/Rh3 subset (arrows). Rh3 (blue) labels pR7s and Rh4 (red) labels yR7s. Bar graphs show GFP co-expression (green) in the Rh3 or Rh4 subset, respectively. Green number indicates the mean percentage of co-expressing photoreceptors, error bar represents standard error of the mean. N = 9 retinas and n = 537 R7s. (B’) The swap of the Rh4 RCSI with the Rh3 RCSI in the Rh4 promoter context does not cause reporter expression in the pR8/Rh5 (blue) yR8/Rh6 (red), or R1-R6 subset. Bar graphs show GFP co-expression (green) in the Rh5, Rh6, or R1-R6 subset. Green numbers indicate the mean percentage of co-expressing photoreceptors, error bar represents standard error of the mean. N = 9 retinas, n = 650 R8s and 3,900 R1-R6 PRs. Scale bars, 10 μm. (TIF) [file pgen.1009613.s003.tif]

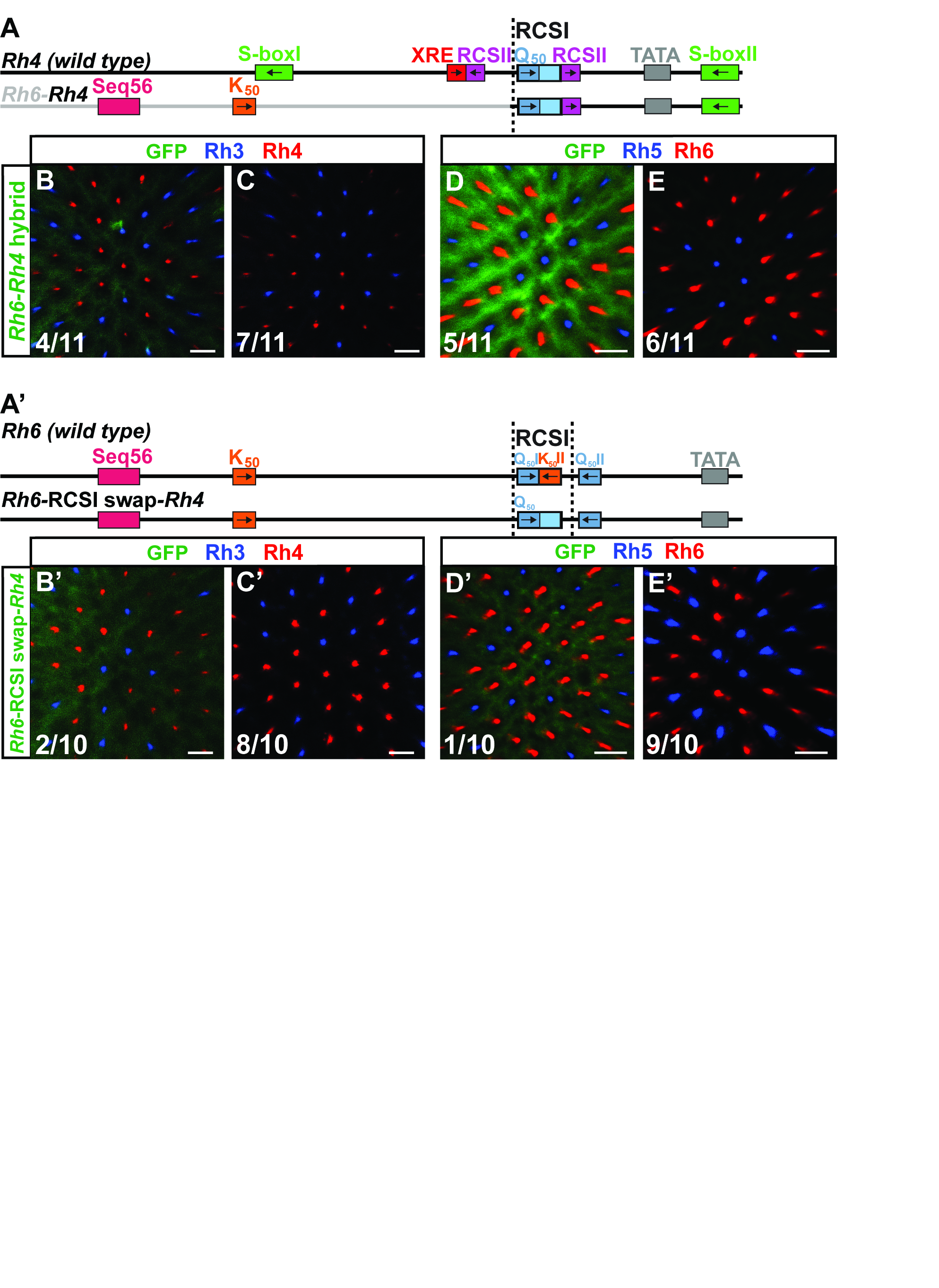

Supplement: S4 Fig — (A) Schematic comparison of the wild type Rh4 promoter and the Rh6-Rh4 hybrid. The dotted vertical line indicates the break/fusion point of the hybrid immediately upstream of the RCSI motif that is found in a similar position in all Rhs. (B)—(C) and (B’)—(C’) Hybrid and RCSI swap driving GFP reporter expression (green) in the R7 layer. Rh3 (blue) labels pR7s and Rh4 (red) labels yR7s. (D)—(E) and (D’)—(E’) Hybrid and RCSI swap driving GFP reporter expression (green) in the R8 layer. Rh5 (blue) labels pR8s and Rh6 (red) labels yR8s. (B) and (C) The Rh6-Rh4 hybrid does not drive detectable GFP expression in R7 photoreceptors. 4/11 retinas exhibit faint reporter expression in pigment cells. N = 11 retinas and n = 1,454 R7s. (D) and (E) The Rh6-Rh4 hybrid does not drive detectable GFP expression in R8 or R1-R6 photoreceptors. 5/11 retinas exhibit reporter expression in pigment cells. N = 11 retinas, n = 830 R8s and 4,980 R1-R6 PRs. (A’) Schematic comparison of the wild type Rh6 promoter and the specific RCSI swap with the Rh4 RCSI (indicated by the dotted vertical lines). Note the shared Q50 motif in the RCSI. (B’) and (C’) The swap of the Rh6 RCSI with the Rh4 RCSI in the Rh6 promoter context does not drive detectable GFP expression in R7 photoreceptors. 2/10 retinas exhibit faint reporter expression in pigment cells. N = 10 retinas and n = 1,089 R7s. (D’) and (E’) The swap of the Rh6 RCSI with the Rh4 RCSI in the Rh6 promoter context does not drive detectable GFP expression in R8 or R1-R6 photoreceptors. 1/10 retinas exhibit faint reporter expression in pigment cells. N = 10 retinas, n = 654 R8s and 3,924 R1-R6 PRs. Scale bars, 10 μm. (TIF) [file pgen.1009613.s004.tif]

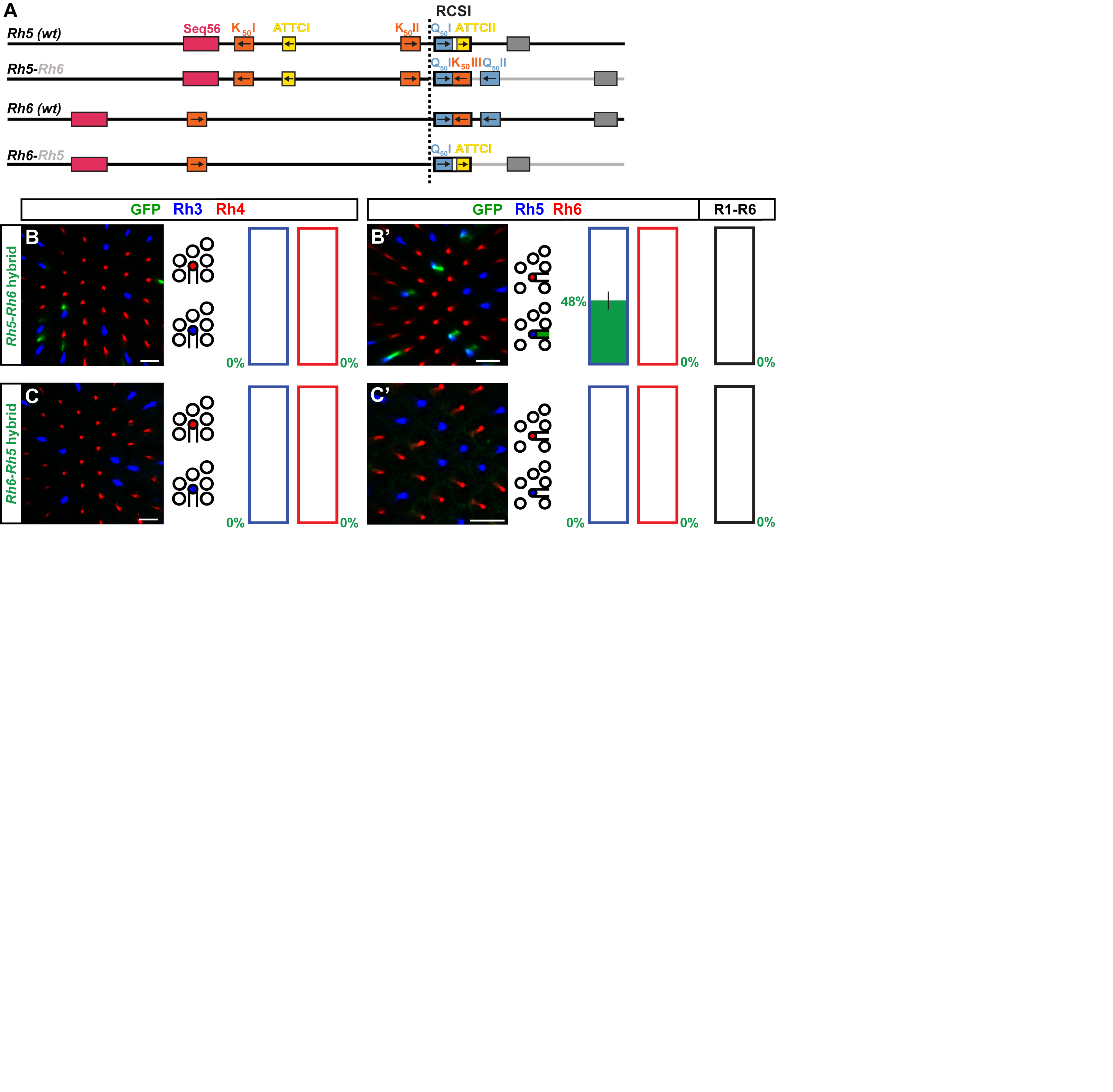

Supplement: S5 Fig — (A) Schematic of wild type (wt) Rh5 and Rh6 promoters and hybrids to test the reverse compatibility of hybrids that had compatible motif combinations (see text). Note the shared K50 motifs and the Seq56 motif. The dotted vertical lines indicate the break- and fusion-points of the hybrids and RCSI swaps. (B) and (C) Hybrid promoter driven GFP reporter expression (green) in the R7 layer. Rh3 (blue) labels pR7s and Rh4 (red) labels yR7s. Bar graphs show GFP co-expression in the Rh3 or Rh4 subset, respectively. Green numbers indicate the mean percentage of co-expressing photoreceptors, error bar represents standard error of the mean. (B’) and (C’) Hybrid promoter driven GFP reporter expression (green) in the R8 layer. Rh5 (blue) labels pR8s and Rh6 (red) labels yR8s. Bar graphs show co-expression of GFP (green) in the Rh5, Rh6, or R1-R6 subset. Green numbers indicate the mean percentage of co-expressing photoreceptors, error bar represents standard error of the mean. (B) and (B’) The Rh5-Rh6 hybrid drives incomplete GFP expression in a fraction of the pR8 subtype. N = 12 retinas and n = 807 R7s for (B); N = 10 retinas, n = 1,364 R8s and 8,184 R1-R6 PRs. (C) and (C’) The Rh6-Rh5 hybrid does not drive detectable GFP reporter expression. N = 12 retinas and n = 858 R7s for (C); N = 8 retinas, n = 773 R8s and 4,638 R1-R6 PRs for (C’). Scale bars, 10 μm. (TIF) [file pgen.1009613.s005.tif]
