# Supplementary material for: A combinatorial cis-regulatory logic restricts color-sensing Rhodopsins to specific photoreceptor subsets in Drosophila
Source: PLoS Genet. 2021 Jun 23;17(6):e1009613. doi: 10.1371/journal.pgen.1009613 (PMC8259978; doi:10.1371/journal.pgen.1009613)
Supplement: S1 Text — (DOCX) [file pgen.1009613.s006.docx]

**S1 Text**

**Similar usage of high-affinity homeodomain motifs in the distal and proximal promoter regions of several *rhodopsins***

The *Rh3*, *Rh5*, and *Rh6* promoters all contain multiple evolutionarily conserved K_50_ homeodomain motifs (TAATCC) (S1B-D Fig). The K_50_ motifs mediate broad activation in all PRs through Otd as well as repression in yR7 and R1-R6 PRs through Dve (Fig 1B). Interestingly, *Rh3*, *Rh5*, and *Rh6* rely on conserved high-affinity homeodomain motif variants [1,2] that are ‘strong’ transcription factor binding sites: of the 80 total K_50_/Otd motif occurrences in 12 sequenced *Drosophila* species (S2B-D Fig), 74 have maximal Otd matrix similarity scores (TAATCC, PWM score: 100; see Materials and methods). *Rh3* has only three occurrences of another high-affinity variant (TAATCT, 84), while *Rh5* has only one (TAATCA, 65) and two (TAATCG, 62) occurrences of slightly lower affinity variants (S2B and S2C Fig) [1,2]. In addition to the K_50_ motif sequence, their position and/or their orientation is also well-conserved, especially in the *Rh6* promoter (S2D Fig). The distal K_50_ motif in the *Rh3* promoter and the *Rh5* promoter shows greater variability than the almost invariant proximal motif (S1B-C and S2B-C Figs); in *Rh5*, the distal K_50_ can be slightly shifted or inverted (S1B and S2B Figs). *Rh6* shows a perfect conservation of both the distal and the proximal K_50_ – that is part of the RCSI – in sequence, position, and orientation (S1D and S2D Figs).

Moreover, the proximal *Rh5* and *Rh6* promoters share perfectly conserved high-affinity Q_50_ motifs that are strong Pph13 binding sites [1,2] (S2C and S2D Fig). All 12 occurrences in *Rh5* have maximal Pph13 matrix similarity scores (TAATTA, 100) (S2C Fig), while all 24 occurrences in *Rh6* of both Q_50_ motifs are of the second highest variant (TAATTG, 90) (S2D Fig). Taken together, the *Rh3*, *Rh5*, and *Rh6* promoters share high-affinity K_50_ and Q_50_ homeodomain motifs, whose arrangements and orientations are evolutionarily conserved.

**Interpretations of the hybrid promoter expression patterns**

Below, we provide more detailed interpretations of the spatial patterns that resulted from the specific combination of distal and proximal *cis*-regulatory motifs in each promoter hybrid. We take advantage of the knowledge of the transcriptional activators and repressors that bind to these motifs (summarized in Fig 10B) [3].

**Hybrid promoter examples for interchangeable core promoters (resulting pattern matches the *Rh* providing the distal promoter region)**

The ***Rh1*-*Rh4*** hybrid drove PR subtype-specific expression in the R1-R6 subset. The replacement of the proximal core promoter in the hybrid retained the *Rh1* RCSI, which is identical with the *Rh4* RCSI, but added the RCSII/yR7 activator motif (that is bound by an unknown factor) [[4](#_ENREF_18)] as well as a single S-box/R8 repressor motif that is bound by Sens [[5](#_ENREF_14)] (Fig 10B). Both motifs do not occur in the *Rh1* promoter, but their presence did not alter the pattern.

The ***Rh3*-*Rh5*** hybrid drove subtype-specific expression in the pR7/Rh3 subset. This hybrid replaced the proximal K_50_/Otd activator/Dve repressor motif in the *Rh3* RCSI with a Q_50_/Pph13 activator motif and removed the proximal Sens/S-box R8 repressor motif. This means added broad activation by Pph13 at the expense of some of the repression in yR7s by Dve and R8s by Sens. Since the outcome was a pR7/Rh3 subset-specific pattern, this suggests that the two remaining distal Dve/K_50_ motifs and Sens/S-box motifs as well as the ATTC/yR7 repressor motif in the RCSI were still sufficient to yield a subtype-specific pattern.

The ***Rh4*-*Rh3*** hybrid drove expression in the yR7/Rh4 subset, just like a wild type *Rh4* promoter. The proximal core promoter from *Rh3* was thus equivalent with the one of *Rh4* and did not interfere with the activation in the yR7/Rh4 subset that is mediated by Ss through the distal XRE motif as well as an unknown factor that binds the RCSII motif (Fig 10B). However, the *Rh3* RCSI in the hybrid theoretically adds conflicting repression in yR7s, where Dve and an unknown repressor bind its K_50_ and ATTC sub-motifs, respectively [[6](#_ENREF_19)]. Since the outcome was expression in yR7s, this suggests that activation prevailed over the potentially conflicting repression. Moreover, the distal XRE/Ss and RCSII motifs clearly can provide combinatorial activation with other proximal motifs in addition to the *Rh4* RCSI and RCSII. Sens (through the S-boxes) and Dve (K_50_ in the RCSI) provide repression in R8s and R1-R6, respectively.

Just like the *Rh4*-*Rh3* hybrid, the ***Rh4*-*Rh5*** hybrid drove expression in the yR7/Rh4 subset. The proximal RCSI from *Rh5* added only one conflicting yR7 repressor motif (ATTC/y) in this hybrid, which was inconsequential for expression in the yR7/Rh4 subset. Note that the *Rh4*-*Rh5* hybrid differs from *Rh4*-*Rh3* in the lack of K_50_ motifs and the presence of a Pph13/Q_50_ motif (Fig 10B).

The fact that both the *Rh4*-*Rh5* and *Rh4*-*Rh3* hybrids were unaffected by conflicting repressor motifs implies that the strength and number of repressor motifs, i.e. whether they are repeated (like in the wild type promoters, Figs 1C and S1A-D), is critical for the resulting *Rh* pattern. Therefore, an alternative explanation for the distal-proximal motif compatibility of these two hybrids is insufficient (combinatorial) repression in the proximal core region as opposed to fully equivalent generic activation.

**Hybrid promoter examples for combinatorial core promoters (resulting pattern does not match the *Rh* providing the distal promoter region)**

The ***Rh4*-*Rh1*** hybrid drove weak expression in all PRs (R1-R8) despite retaining the RCSI motif, which is the same in *Rh4* and *Rh1*. The striking difference to the subtype-specific *Rh4*-*Rh3* and *Rh4*-*Rh5* hybrids is thus caused by motifs or other features in the proximal *Rh1* region downstream of the RCSI.

The ***Rh4*-*Rh6*** hybrid drove variable but also relatively broad expression. However, it did not label pR7s and pR8s. As the *Rh4*-*Rh6* contains a high-affinity K_50_/Otd activator/Dve repressor motif in the RCSI that is absent in *Rh4*-*Rh1*, the variable expression in R1-R6 could be due to partial repression mediated by the high levels of Dve in R1-R6 [[7](#_ENREF_12)]. The lack of expression in pR7s or pR8s could be caused by unknown repressor motifs in the proximal *Rh6* promoter.

The ***Rh3*-*Rh4*** hybrid drove ‘pan-R7’ expression in both the pR7/Rh3 subset and the yR7/Rh4 subset. It replaced the proximal yR7 repressor motifs (K_50_/Dve and ATTC) from *Rh3* with activation in the yR7/Rh4 subset by the *Rh4* RCSI and RCSII motif [[6](#_ENREF_19)] (Fig 10B). This suggests that distal and proximal motifs contributed to the expression pattern, which resembles an addition of both contributing promoters.

The ***Rh3*-*Rh6*** hybrid drove broadened, ‘pan-inner’ PR expression in both R7 subtypes and in both R8 subtypes. The major differences to the pan-R7 hybrid *Rh3*-*Rh4* are two Q_50_/Pph13 activator motifs instead of one, three K_50_/Otd activator/Dve repressor motifs instead of two, and two instead of three S-box/Sens/R8 repressor motifs (Fig 10B). The addition of a second, strongly activating high-affinity Q_50_/Pph13 motif from *Rh6* appears to have overcome the repression in yR7s, where Dve is only expressed at low levels. The expansion into R8 PRs was likely due to the loss of the proximal S-box R8 repressor motif.

The ***Rh5*-*Rh4*** hybrid drove expression in the pR8/Rh5 subset, as expected from the distal *Rh5* region, but surprisingly also in the pR7/Rh3 subset. The hybrid pattern is therefore ‘p’-subtype restricted. The expression in the pR8/Rh5 and pR7/Rh3 subset is likely due to the presence of two K_50_ motifs through which Otd activates in R1-R8 and Dve represses in yR7s and R1-R6 (Fig 10B), but this does not explain why the proximal region from *Rh4* – but not *Rh3* (see *Rh5*-*Rh3* below) – caused activation/misexpression in pR7s. One reason could be additional activation by the high-affinity Q_50_/Pph13 motif that is missing in the proximal *Rh3* promoter.

The ***Rh5*-*Rh6*** hybrid drove partial activation in the pR8/Rh5 subtype, which could either be due to a pR8 repressor motif in the proximal *Rh6* promoter or other features that do not support activation in pR8s.

The ***Rh5*-*Rh3*** hybrid surprisingly did not drive expression in PRs, suggesting that the distal and proximal part were incompatible despite similar proximal promoters (3’ RCSI motifs and spacing to the TATA box).

The ***Rh6*-*Rh3*** hybrid drove variable expression in some PRs of the yR8/Rh6 subset and the R1-R6 subset. Since specifically swapping the *Rh6* RCSI with the *Rh3* RCSI in the *Rh6* promoter context caused derepression in yR7s and some pR7s, motifs or features downstream of the *Rh3* RCSI must have been responsible for the derepression in R1-R6. The much stronger expression in R8s in the RCSI swap experiment could be due to the lack of the proximal Sens/S-box R8 repressor motif that is present in the proximal region of *Rh3*.

The ***Rh6*-*Rh4*** hybrid did not drive expression in photoreceptors, which appears to be due to the incompatibility of the distal *Rh6* region with the *Rh4* RCSI that lacks the 3’ ATTA core repeat of the *Rh6* RCSI.

The ***Rh6-Rh5*** hybrid also failed to drive detectable reporter expression. Since we previously showed that the specific RCSI swap and the addition of one ATTC/yR8 repressor motif only mildly affect activation in yR8s [[6](#_ENREF_19)], other features downstream of the *Rh5* RCSI, such as the close spacing to the TATA, were incompatible with activation in yR8s.

**References**

1. Zhu LJ, Christensen RG, Kazemian M, Hull CJ, Enuameh MS, Basciotta MD, et al. (2011) FlyFactorSurvey: a database of Drosophila transcription factor binding specificities determined using the bacterial one-hybrid system. Nucleic Acids Res 39: D111-117.

2. Noyes MB, Christensen RG, Wakabayashi A, Stormo GD, Brodsky MH, Wolfe SA (2008) Analysis of homeodomain specificities allows the family-wide prediction of preferred recognition sites. Cell 133: 1277-1289.

3. Rister J, Desplan C, Vasiliauskas D (2013) Establishing and maintaining gene expression patterns: insights from sensory receptor patterning. Development 140: 493-503.

4. Fortini ME, Rubin GM (1990) Analysis of cis-acting requirements of the Rh3 and Rh4 genes reveals a bipartite organization to rhodopsin promoters in Drosophila melanogaster. Genes Dev 4: 444-463.

5. Xie B, Charlton-Perkins M, McDonald E, Gebelein B, Cook T (2007) Senseless functions as a molecular switch for color photoreceptor differentiation in Drosophila. Development 134: 4243-4253.

6. Rister J, Razzaq A, Boodram P, Desai N, Tsanis C, Chen H, et al. (2015) Single-base pair differences in a shared motif determine differential Rhodopsin expression. Science 350: 1258-1261.

7. Johnston RJ, Jr., Otake Y, Sood P, Vogt N, Behnia R, Vasiliauskas D, et al. (2011) Interlocked feedforward loops control cell-type-specific Rhodopsin expression in the Drosophila eye. Cell 145: 956-968.
